# GSK3 as a Regulator of Cytoskeleton Architecture: Consequences for Health and Disease

**DOI:** 10.3390/cells10082092

**Published:** 2021-08-14

**Authors:** Daria Hajka, Bartosz Budziak, Łukasz Pietras, Przemysław Duda, James A. McCubrey, Agnieszka Gizak

**Affiliations:** 1 Department of Molecular Physiology and Neurobiology, University of Wrocław, Sienkiewicza 21 Street, 50-335 Wrocław, Poland; daria.hajka@uwr.edu.pl (D.H.); bartosz.budziak@uwr.edu.pl (B.B.); lukasz.pietras2@uwr.edu.pl (Ł.P.); przemyslaw.duda@uwr.edu.pl (P.D.); 2 Department of Microbiology and Immunology, Brody School of Medicine at East Carolina University, 600 Moye Boulevard, Greenville, NC 27858, USA; mccubreyj@ecu.edu

**Keywords:** glycogen synthase kinase-3, actin, tubulin, cytoskeleton, brain development, cancer cells migration, mitochondria trafficking

## Abstract

Glycogen synthase kinase 3 (GSK3) was initially isolated as a critical protein in energy metabolism. However, subsequent studies indicate that GSK-3 is a multi-tasking kinase that links numerous signaling pathways in a cell and plays a vital role in the regulation of many aspects of cellular physiology. As a regulator of actin and tubulin cytoskeleton, GSK3 influences processes of cell polarization, interaction with the extracellular matrix, and directional migration of cells and their organelles during the growth and development of an animal organism. In this review, the roles of GSK3–cytoskeleton interactions in brain development and pathology, migration of healthy and cancer cells, and in cellular trafficking of mitochondria will be discussed.

## 1. Introduction

Glycogen synthase kinase-3 (GSK3) is a serine/threonine kinase. It was initially identified as a regulator (inhibitor) of glycogen synthesis [1]. It has since been recognized as a multifunctional kinase with a variety of roles both in invertebrates and in vertebrate cells. In mammals (humans included), there are two closely related isoforms of the kinase: GSK3α and GSK3β [2]. They share ~98% identity in their kinase domains, but they have distinct substrate preferences, and their cellular functions are, at least partially, non-redundant [3,4]. The β isoform predominates in the majority of cells, and it is also more studied. GSK3 requires phosphorylation on tyrosine 216 (β isoform) or 279 (α isoform) for maximal activity. As a constitutively active kinase, it is oftentimes inhibited in response to upstream signals by phosphorylation of S9 (GSK3β) or S21 (GSK3βα). As a sensor of growth factors (e.g., insulin, transforming growth factor-β, epidermal growth factor, nerve growth factor, and brain-derived neurotropic factor) and other extracellular stimuli GSK3 is a master switch kinase regulating various aspects of cellular function such as growth, repair, mobility, and survival. Therefore, it is not surprising that dysregulation of GSK3 activity is observed in many pathophysiological processes, including the development of cancer, and neurodegenerative and psychiatric disorders (for review see [5,6]). This also makes GSK3 an attractive therapeutic target, and intensive efforts have been undertaken to discover clinically relevant selective GSK3 inhibitors [7]. However, the pleiotropic functions of the kinase pose major obstacles in developing effective treatments without adverse effects.

In the still-increasing list of GSK3 substrates, there are, among others, proteins engaged in the regulation of actin cytoskeleton dynamics (e.g., Rho family members and related GTPases), microtubule-associated proteins (MAPs, e.g., Tau and collapsin response mediator protein 2 (CRMP 2)) and adhesion of cells to extracellular matrix (e.g., focal adhesion kinase, FAK) [8,9,10]. Acting through these substrates, GSK3 can influence cell polarization and directional migration, as well as intracellular trafficking of mitochondria and vesicular structures. The migration of cells is a fundamental process, especially important in the development of an organism. Although not all differentiated adult cell types migrate in vivo, almost all of them exhibit some form of spatial segregation of structures and functions and require some form of organelles’ trafficking. This, in turn, requires the orchestration of activities of several signaling pathways. Since GSK3 lies at the crossroads of these pathways, it can be viewed as one of the coordinators/integrators of complex processes of cellular dynamics.

This review discusses the key roles of GSK3-to-cytoskeleton and GSK3-to-extracellular matrix signaling in the brain, cardiac, and cancer cells, in normal and pathological settings.

## 2. GSK3–Cytoskeleton Interplay in Brain Development and Pathology

GSK3β is expressed in all tissues, including the brain [2]. In the rodent central nervous system (CNS), GSK3β is more abundantly expressed than GSK3α, and thus, it is also better studied. This isoform is involved in numerous events during neurogenesis, synaptic plasticity, and neurodegeneration (for review see [6,11]). During brain development, GSK3β is highly expressed in neurons and barely detectable in astrocytes [12,13]. In rodent embryos, GSK3β is detected in axons, perikarya, and the proximal part of dendrites of postmitotic neurons (in neuroblasts, the kinase is hardly detectable), but after the 10th day of postnatal life, it starts to disappear from the axonal tracts. Globally, the expression of GSK3β in the brain is supposed to be higher in rodent embryos and in early postnatal life, than in adults, positively correlating with the major period of dendritic extension and synaptogenesis [12], although it has been suggested that in the murine brain (hippocampus, cerebral cortex, and cerebellum), such age-related changes applied to proteins comprising the “GSK3 proteome” rather than the GSK3 (α and β) protein level [14]. The expression of GSK3β increases again later in aged (24–29-month-old) rodent brains [15]. This increase is correlated with the development of neurodegenerative diseases such as Alzheimer’s disease (AD) and thus is a promising target of a future anti-neurodegenerative therapy.

Substrates of GSK3β can be divided into three groups: metabolic/signaling proteins (e.g., acetyl-CoA carboxylase, pyruvate dehydrogenase, glycogen synthase, insulin receptor substrate-1, amyloid-beta precursor protein, cyclin D1, protein phosphatase 1); structural proteins (e.g., dynamin-like proteins, microtubule-associated protein 1B and 2 (MAP1B and MAP2), neural cell-adhesion protein, neurofilaments, spindle-associated protein Astrin, and microtubule-associated protein Tau), and transcription factors (e.g., activator protein 1 (AP-1), cAMP response element-binding protein, glucocorticoid receptor, Myc, nuclear factor of activated T cells and nuclear factor kappa-light-chain-enhancer of activated B cells (NF-κB)) [11]. Due to the long list of substrates, GSK3β can modulate numerous signaling pathways both directly and indirectly, leading to cytoskeleton remodeling. In turn, proper cytoskeleton maintenance and remodeling are indispensable for the correct development and functioning of the CNS.

### 2.1. Neurogenesis

#### 2.1.1. Proliferation

It has been suggested that overexpression or high activity of GSK3β in developing brain negatively regulated proliferation of neural precursors, leading to microcephaly [16,17]. In turn, inhibition of GSK3β (with either lithium or SB216763) promoted the proliferation of cerebellar granulate neuron progenitors [18] and hippocampal neurogenesis [19]. On the other hand, some studies have shown that GSK3β activity was necessary for cell cycle progression, while inhibition of GSK3β with SB216763 led to neural differentiation [20]. It has been also shown that forced activation of GSK3β promoted the expression of cyclin D1, cyclin E, transcription factor E2F-1, and the phosphorylation of retinoblastoma protein [21], which were engaged in cell cycle progression. In addition to affecting the expression of signaling proteins, GSK3β has also regulated spindle microtubule (MT) assembly by interaction with spindle-associated protein Astrin [22]. Astrin plays an important role in chromosomal alignment during metaphase. Both Astrin and GSK3β have been shown to co-localize with the spindle apparatus during mitosis and affect spindle organization during cell cycle progression [23,24]. The kinase activity of GSK3β was indispensable for the interaction of Astrin with microtubules and kinetochore. Therefore, inhibition of GSK3β led to chromosomal aberrations [22]. Furthermore, MT minus end-anchoring protein Ninein, which is engaged in astral microtubules formation during cell division, is also a substrate for GSK3β. Under conditions of low GSK3β activity, Ninein accumulated around mature centrioles what promoted cell division and preservation of progenitor characteristics of cells. In turn, the high activity of GSK3β resulted in the lack of Ninein accumulation in centrosomes and caused differentiation of progenitors to become intermediate progenitor cells or neurons [25,26]. GSK3β can also regulate the activity of mitogenic transcription factors: AP-1 and NF-κB [27,28]. AP-1 regulates differentiation, proliferation, and apoptosis by activation of Rho-like GTPases [29]. Rho proteins play a fundamental role in many cellular processes, such as cellular movement, establishing cell polarity, mitosis [30], and nervous system reorganization during growth and injury [31,32] by influencing actin cytoskeleton dynamics. NF-κB controls many inflammatory processes, cell proliferation, differentiation, and survival [33]. Active NF-κB has correlated with cytoskeleton rearrangements, but GSK3β negatively regulated NF-κB in neurons and astrocytes [33,34].

**Figure 1:**
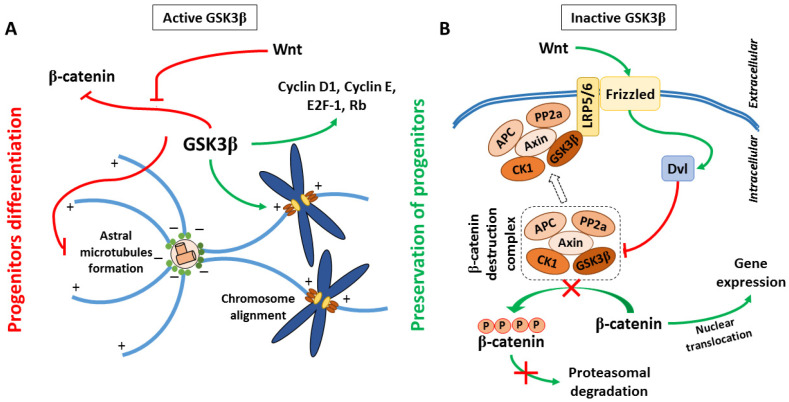
Proposed roles of GSK3β in the regulation of proliferative potential of neural precursors: (**A**) GSK3β activity sustains progenitor characteristics of cells by regulation of chromosomal alignment along the metaphase plate, and promotion of cyclins and transcriptional factors expression. On the other hand, GSK3β inhibits the formation of astral microtubules and directs β-catenin to degradation, promoting differentiation of cells (the microtubule plus- and minus-ends are indicated as + and −, respectively); (**B**) GSK3β is a part of the “β-catenin destruction complex”. The complex phosphorylates β-catenin what leads to proteasomal degradation of this transcription factor. Wnt signaling inactivates the complex acting via Disheveled (Dvl) protein. This enables nuclear translocation of β-catenin, which enhances the proliferation of neural precursors.

Furthermore, it has been demonstrated that inhibition of GSK3β caused β-catenin nuclear accumulation, which enhanced the proliferation of neural precursors [35]. β-catenin is a component of Wnt signaling. When the Wnt pathway is turned off, the destruction complex consisting of Axin, adenomatous polyposis coli (APC), protein phosphatase 2A, GSK3, and Casein kinase 1α [36,37] results in β-catenin degradation by its ubiquitination and targeting to the proteasome [38,39]. However, when some proteins from the Wnt family were bound to Frizzled/LRP 5/6 co-receptor, the destruction complex was disabled and migrated to the plasma membrane, where Axin bound to the cytoplasmic tail of LRP 5/6. Meanwhile, the Disheveled (Dvl) protein was activated by the receptor and inhibited GSK3β in the destruction complex, which caused axon MT stabilization [40]. This led to the accumulation of β-catenin in the cytoplasm and its migration to the nucleus, where it triggered T cell factor/lymphoid enhancing factor transcription (TCF/LEF), which regulated the process of proliferation and differentiation [41]. Additionally, APC can bind to the plus ends of MTs during cell division, when GSK3β is inhibited. APC anchors astral MTs to the cell cortex or spindle MTs to the kinetochore, and it is especially important for asymmetrical stem cells division. In summary, maintaining the proper level of expression and activity of GSK3β during subsequent developmental stages of CNS is necessary for proper progress of cell division and differentiation. The proposed roles of GSK3 in the regulation of neurogenesis are presented in Figure 1. Additionally, for the convenience of the reader, information about main GSK3 targets and upstream regulators mentioned in the present paper, their cellular roles, major effects of the GSK3-target protein interactions, together with the experimental model and methods of detection used in the reviewed papers, has been summarized in the Supplementary Tables S1 and S2.

#### 2.1.2. Migration

Directional movement of neural cells is crucial for proper CNS development. The migration of cells is regulated by extracellular signals and achieved by the reorganization of actin filaments and microtubules. It has been shown that it is activated in response to the oscillation of trophic factors [42], GSK3β was an important linker between these signals and cytoskeleton assembly [43,44]. Deficiency in expression or lack of phosphorylation of MAP1B by GSK3β led to abnormalities during brain development. MAP1B regulates actin and MT dynamics, which caused cytoskeletal rearrangements during cell migration [45,46], axonal growth, and regeneration [47,48]. GSK3β also phosphorylates FAK. As the name suggests, FAK is involved in the regulation of cell adhesion, proper spreading, and migration [10]. Lack of FAK expression is manifested by lower adhesion strength, increased cytoskeletal dynamics (enhanced cortical actin distribution), and reduction in cell spreading [49]. Activated (phosphorylated) FAK is then recruited to focal adhesions [50] to promote turnover of cell contacts with an extracellular matrix, which manifests itself as cell migration.

Another key feature of cells, often but not always associated with their migratory potential, is polarity, i.e., intrinsic asymmetry in the structure and organization of molecules and organelles within a cell. In astrocytes, polarity has been shown to be controlled by Cdc42 acting through Par6–PKCζ to phosphorylate GSK3β. This phosphorylation influenced polarization of the centrosome—the major microtubule-organizing center of a cell (MTOC)—and thus, controlled the direction of astrocytic protrusions by inducing interactions of APC with the plus ends of microtubules [51].

S100B protein, a member of a family of Ca^2+^-binding proteins of the EF-hand type, is highly expressed in astrocytes, and it regulates the assembly of MTs and intermediate filaments. Silencing of the protein expression in Muller glial cells and astrocytoma has been shown to lead to inhibition of the PI3K/Akt pathway and, in consequence, to GSK3β activation. This resulted in actomyosin stress fibers disassembly, cortical localization of F-actin, change of morphology of the cells (stellation of astrocytes), and reduction in their migration. This suggested that GSK3β, acting through cytoskeleton, was a part of the mechanism regulating invasiveness of glioma cells, migration of astrocytes to places of brain insult, and also differentiation of the cells [52].

#### 2.1.3. Differentiation

During CNS development, future neurons should undergo multiple rearrangements to create highly specialized cells. GSK3β is engaged in processes of neuronal polarization, axonal growth, and branching by regulating Wnt signaling and by interacting with numerous microtubule-binding proteins (MBPs). The first step in neuronal differentiation is breaking the cell symmetry and triggering cytoskeleton remodeling to create future axon and somatodendritic compartments. This can be achieved by asymmetrical activation of intracellular signals, which is caused by activation of several signaling pathways: PI3K, Rho-GTPase, Par3/6 pathway, TSC-mTOR, and PKA-LKB1 pathway. GSK3β is known to have an impact on all these pathways, which makes it an important modulator during neuronal differentiation [53,54,55,56].

**Figure 2:**
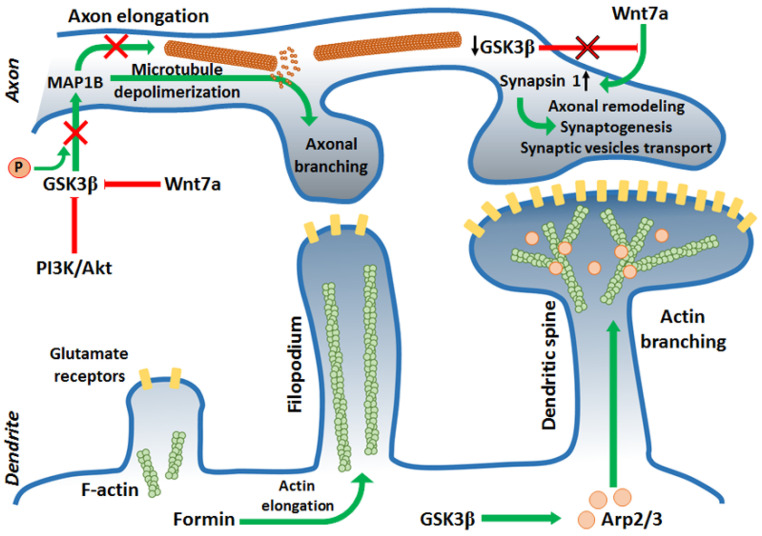
GSK3β is engaged in synaptogenesis by regulation of maturation of pre- and postsynaptic termini. In the axon, GSK3β regulates the stabilization and assembly of microtubules by interaction with and phosphorylation of several microtubule-binding proteins (e.g., MAP1B). Activation of GSK3β stimulates axonal elongation, whereas inhibition of the kinase promotes axonal branching. Constitutive activity of GSK3 leads to the formation of multiple axons thus, the PI3K/Akt pathway activation is indispensable to achieve proper neuronal polarity during cell differentiation. Active GSK3β also inhibits the Wnt signaling pathway, which results in the reduction in Synapsin 1 expression. Thus, inhibition of GSK3β leads to Wnt- and Synapsin 1-dependent axonal remodeling and synaptogenesis. In dendrites, GSK3β activates Arp2/3 protein, which is responsible for actin filaments branching. Thus, GSK3β participates in the transformation of filopodia into dendritic spines.

PI3K/Akt pathway activity is indispensable for cytoskeleton rearrangement and phenotype switching during cell differentiation [57]. GSK3β, positioned downstream of PI3K/Akt, must be inhibited by Akt to achieve and maintain proper neuronal polarity (Figure 2). It has been shown that GSK3β inhibition with SB216763, SB415286, or lithium led to the formation of multiple axons from preexisting dendrites, while the constitutively active form of the kinase resulted in the lack of axons [58,59].

After establishing the future axon location, further actin filaments and MT rearrangements are required. At this stage, GSK3β has been shown to interact with and phosphorylate numerous MBPs: collapsin response-associated protein (CRMP2) [60], APC, MAP1B [44], whose main task was stabilization and assembly of MTs. Dephosphorylated APC interacted with plus ends of MTs leading to increased stabilization of MTs and microtubules–actin interactions during axon elongation [61]. Dephosphorylated CRMP2 interacted with tubulin heterodimers promoting MTs assembly during axon specification and branching. Inhibition of GSK3β activity was sufficient to initiate cytoskeleton rearrangements regulated by these proteins. In turn, phosphorylation of MAP1B, a protein promoting elongation in primary axon by GSK3β led to activation of this MBS. Inhibition of GSK3β activity reduced the amount of phosphorylated MAP1B and thus, mitigated microtubule dynamics [61] (Figure 2). This points to a complex mechanism of cytoskeleton dynamics regulation that requires a precise balance between activation and inhibition of GSK3.

Certain polarity-inducing signals have inhibited GSK3β and allowed MT stabilization and axon elongation [59,62,63]. However, it has been shown that the reduction of GSK3 activity should be “localized” and restricted to the axonal growth cone. Global inhibition of GSK3 caused axonal growth difficulties because of a reduced pool of dynamic microtubules at the growth cone that led to excessive MT stability [61]. Finally, after the axon elongates enough, it starts branching to innervate multiple targets. Once again, a proper level of GSK3β activity reduction (creating a kind of “intermediate conditions” that preserve a pool of dynamic microtubules) is needed for this process [61]. C-Type Natriuretic Peptide, cGMP, and cGMP-dependent protein kinase G1 are known to inhibit GSK3β activity at this point [64,65], but Wnt signaling also seems to be involved [66]. Additionally, JNK-interaction protein 3 (JIP3) can restrict axonal branching by interaction with GSK3β. Knocking down JIP3 or GSK3β increases axon branching [67]. Furthermore, PTEN, which dephosphorylates phosphatidylinositol (3,4,5)-triphosphate (PIP3) and decreases PI3K signaling is known to negatively regulate axonal branching. It has been proposed that during induction of the branching, downregulation of PTEN was caused by GSK3β inactivation [68,69]. Similar to axonal elongation, during axonal branching, the F-actin reorganization and MT extension occur [70]. However, it is not fully understood how molecular regulation of GSK3β is translated into axonal branching mechanisms, although it is known that local inactivation of GSK3β along the axon promotes cytoskeletal reorganization required for the process.

### 2.2. Synaptic Plasticity

GSK3β can phosphorylate several proteins involved in cytoskeleton reorganization needed for morphological changes in synapses (synaptic plasticity) during memory consolidation. Due to synaptic plasticity, the connections between two neurons are either strengthened (in the process of long-term potentiation, LTP) or weakened (long-term depression, LTD) in response to increases or decreases of neuronal activity. Synaptic plasticity is a prerequisite to learning. The connection between two neurons can be strengthened by the recruitment of neurotransmitters’ receptors to the membrane of the postsynaptic neuron [71] or by releasing higher amounts of neurotransmitters to the synaptic cleft by the presynaptic neuron [72]. In turn, the connection is weakened by retraction of the receptors and decreased neurotransmitter secretion. Some cytoskeletal changes occur during dendritic spine maturation. During strengthening of the connections, dendritic spines start to grow in volume creating mushroom-shaped outgrowth, the spines can also branch. This leads to increased surface area in the postsynaptic membrane, creating the possibility to incorporate more receptors into the membrane [73]. It has been demonstrated that during the connection strengthening, dendritic filopodia (precursors of the spines) start to elongate. In this process, Formin (an actin regulatory protein) promoted elongation of unbranched actin filaments. Additionally, Actin-related protein 2/3 complex (Arp2/3) actions led to branched F-actin networks formation. Arp2/3 bound to the existing F-actin filament and initiated a new filament creation with a 70-degree angle. This led to an increased pool of stable F-actin in the center zone of the dendritic spine, and a more branched dynamic F-actin pool in the peripheral zone, which resulted in volume expansion of the dendritic spine [74,75]. Inhibition of GSK3 with SB415286 or LiCl2 prevented the accumulation of Arp2/3 in the cellular extensions [76] (Figure 2).

Wnt signaling is, among others, responsible for synapse formation and differentiation [77]. A protein in the Wnt family, Wnt7a, has been shown to play an important role in axon remodeling and recruiting synaptic receptors during the connection strengthening [77,78] (Figure 2). Wnt7a promoted MT depolymerization by decreasing MAP1B phosphorylation, which led to axonal spreading [78,79]. Additionally, Wnt7a increased the level of Synapsin I, which was engaged in axonal remodeling, synaptogenesis, and transport of synaptic vesicles to the growing area [79,80,81], but the mode of interaction between Synapsin I and cytoskeleton during axon and synapse remodeling remains unknown. To activate Wnt signaling, GSK3β must be inhibited. Inhibition of GSK3β caused, therefore, more abundant axonal branching [79,82] and promote LTP maintenance [83,84], while activation/overexpression of GSK3β disturbed LTP formation and led to LTD by decreasing synaptic transmission and secretion of neurotransmitters in a Synapsin I-dependent manner [83,84,85] and downregulation of the NR2A/B subunit of NMDA receptor (N-methyl-D-aspartate receptor) in postsynaptic neurons [83]. Inhibition of PI3K or Wnt signaling pathways caused LTP disruption, because of GSK3β activation [86,87]. On the other hand, GSK3β activity was indispensable for synaptic vesicles recycling during neuronal activity. The phosphorylation of Dynamin I either by GSK3β or cyclin-dependent kinase 5 caused endocytosis of synaptic vesicles [88]. Dynamin I interacts with both MTs and actin filaments regulating their dynamics [89] since vesicles endocytosis requires actin cytoskeleton rearrangements [90].

### 2.3. Neurodegenerative Disorders and Neuronal Survival

Apoptosis is necessary for proper CNS development—almost half of the immature neurons die in a process of programmed cell death before CNS is matured completely [91]. On the other hand, increased apoptosis occurs during neurodegenerative events [92]. It has been shown that GSK3β played a dual role in apoptosis promotion, depending on the organism’s developmental stage. Inhibition of GSK3β (with lithium) promoted apoptosis in immature neurons, while in mature neurons, it supported cell survival [93]. In vivo, GSK3β can be activated in response to environmental conditions fluctuations, lack of trophic factors, cellular stress (oxidative, endoplasmic, DNA damage), which leads to increased apoptosis in mature cells [94,95,96]. GSK3β can induce apoptosis in two ways—by phosphorylation of microtubule-associated protein Tau, which leads to MT destabilization and cytoskeleton collapse [97], or by interaction with several transcription factors and proapoptotic proteins. Tau interacts with MTs maintaining its dynamics. The presence of hyperphosphorylated Tau protein is characteristic of neurodegenerative processes and causes the formation of Tau aggregates, which leads to MT disorganization that is correlated with apoptotic cell death induction [97].

During brain injury or infection-induced inflammation, microglia motility increases to facilitate phagocytosis of damaged cells. GSK3β activity has also a large impact on these processes (for review see [98]). GSK3β inhibition (with lithium, indirubin-3′-monoxime (I3′M), or kenpaullone) has been shown to attenuate the response of microglia to pro-inflammatory stimuli and resulted in a marked reduction of their motility–both random and chemotactically directed, without more general impairment of microglia functions [99]. As neurodegenerative changes in the brain are often the result of long-lasting or prolonged microglia activation, mitigation of GSK3β activity might be considered a means to limit neuroinflammation and to stave off the effects of age-related diseases, such as AD [100]. In AD, amyloid-β plaques are sites of accumulation of reactive microglia but also astrocytes. Astrocytes secrete signals that further promote microglia activation and migration. It has been shown that GSK3β phosphorylation of the transcription factor CCAAT/enhancer-binding protein delta (CEBPD) in astrocytes was responsible for these processes, and that inhibition of GSK3β by LiCl attenuated murine microglia migration [101]. Although the authors of the cited paper seemed to suggest that chemotaxis was the main cause of the observed phenomenon, the participation of GSK3 in the reorganization of the cytoskeletal structure during migration-related processes–from the initiation of polarization, through the extension of cellular processes to the modulation of cell adhesion–indicates that it is not the only cause.

To summarize, since neuroinflammation contributes to the initiation and intensification of numerous pathological conditions, controlling the effects of GSK3β on cytoskeleton architecture might be beneficial in a broad array of neurodegenerative diseases.

## 3. GSK3–Cytoskeleton Interplay in Cell Motility and Migration of Cancer Cells

Cell motility is a random process of cell movement, while cell migration plays a role in organ and tissue formation, but it is also used by cancer cells to spread in a process known as metastasis (for review see [102]).

The role of GSK3 in cell migration and motility is still controversial, and although most studies have demonstrated that active GSK3 stimulated these processes [76,103,104,105,106], there are also reports indicating that active GSK3 inhibited cell movement [107,108,109].

Cell movement depends on the activity of two factors regulating antagonistic modes of cell migration, the mesenchymal (driven by Rac1-GTPase) and amoeboid (driven by RhoA-GTPase) mode. Rac1 downregulates RhoA expression via p190RhoGAP and PAK/GEF-H1 and, reciprocally, RhoA downregulates Rac1 via ROCK/FilGAP and ROCK/ARHGAP22. During the mesenchymal mode, the movement is driven by the formation of actin-rich protrusions called lamellipodia, and cells interact with the extracellular matrix (ECM) through focal adhesions (FAs). The amoeboid mode is driven by protrusions called blebs and it is FA independent. Cells undergoing amoeboid movement are characterized by rounded cell morphology than cells using the mesenchymal mechanism (for review see [110,111]).

Cancer cells exhibit various modes of migration. For example, U87MG glioblastoma cells are strictly mesenchymal mode cells, and Rac1 signaling inhibition blocks their movement [112]. In other mesenchymal mode cells, the HT1080 fibro-sarcoma, suppression of Rac1 signaling does not inhibit the movement but leads to mesenchymal-to-amoeboid transition. Subsequent inhibition of RhoA signaling entirely blocks their motility. In turn, the amoeboid SW480 human colon adenocarcinoma cells adopt mesenchymal phenotype after RhoA or ROCK inhibition [112].

Although a direct effect of GSK3 on the mechanisms of cell migration has not been studied, there are numerous references documenting the effects of GSK3 on the activity of essential proteins involved in cell movement.

### 3.1. Lamellipodia, Filopodia, and Invadopodia Formation and Dynamics

Lamellipodia are cell protrusions formed by branching actin filaments, which induce forward movement of the cell membrane during the mesenchymal mode of migration. In this mode of movement, Rac1 activates actin filaments branching complex Arp2/3 in lamellipodia. Continuous branching of the filaments creates forces that overcome the tension of the cell membrane, which, in turn, allows forward movement of the protrusion (for review see [110,111]). Activation of Arp2/3 by Rac1 is mediated by Wiskott–Aldrich syndrome protein family member 2 (WASF2) [113]. It has been shown that GSK3 regulated indirectly the activity of Rac1 [103], and thus, the migration of cells. Inhibition of GSK3 with the inhibitor IX and SB415286 blocked the activity of Rac1 upstream effector ADP-ribosylation factor 6 (ARF6) and reduced lamellipodia extension and cell migration [103]. The expression of constitutively active ARF6 prevented the effects of GSK3 inhibition [103], suggesting that GSK3 regulated Rac1 via ARF6. Additionally, it has been shown that in human glioblastoma cell lines (T98G and U87), the GSK3 inhibitor AR-A01441 decreased the expression of several Rac1 activating proteins [104]. The study suggested that GSK3 exerted long-term effects on cell motility by sensitizing the molecular machinery prerequisite for cell directional movement.

Another line of evidence of participation of GSK3 in the Rac1-dependent lamellipodia formation has emerged from studies on human keratinocyte cell line HaCaT and the Rat2 fibroblasts [76,114] that showed that GSK3 stimulated Rac1 and, consequently, Arp2/3 activity. In the HaCaT cells, inhibition of GSK3 with lithium or SB415286 blocked Rac1 and Arp2/3 localization in lamellipodia ruffles [76,114]. In the Rat2 fibroblasts, GSK3β inhibition with synthetic triterpenoids CDDO-Im or CDDO-Me was correlated with a decrease in the Arp2/3-dependent actin branching and reduced lamellipodia formation [114,115]. Additionally, overexpression of proteins from the Rho family of GTPases (RhoA and Cdc42) prevented the formation of the long lamellipodia (the extended lamellipodia, E-lam) in the keratinocyte cell line [76]. Activation of these proteins has been shown to lead to phosphorylation and inhibition of GSK3β through PKC [51].

GSK3β activity may also lead to activation of cofilin, an actin rearrangement factor [116]. Cofilin is downstream of the Rac1/Wiskott-Aldrich syndrome protein family mem-ber 2 (WASF2)/Arp2/3 pathway. It has been demonstrated that downregulation of this pathway reduced the human glioma cell line U251 migration [113]. In human breast adenocarcinoma cell line MDA-MB-231, inhibition of GSK3β by AR-A0114418 decreased migration and actin branching via WASF2 downregulation [117]. Furthermore, inhibition of GSK3 in the human myeloid leukemia U937 cell line with lithium or I3′M resulted in Rac1 inhibition and, consequently, inactivation of cofilin, which disturbed lamellipodia formation [116]. It has been shown that silencing of GSK3β or its inhibition with AR-A01441 reduced the migration of human glioblastoma cell lines: U87, U251, A172, and T98G [105]. The inhibition also reduced the number of lamellipodia and the localization of Rac1 and actin filaments in lamellipodia [104]. Similar effects have been observed after GSK3β inhibition by AR-A01441 in human pancreatic cancer cell lines: MIA PaCa-2 and PANC-1 [105].

On the other hand, the presence of a negative feedback mechanism between Rac1 and GSK3 has been suggested [118]. In the human non-small cell lung carcinoma cell lines (HCC44, H2122, H358, HTB55, Colo699, H1299, A549), activated Rac1 stimulated indirectly (via P21 Activated Kinase, PAK1) Akt kinase, which, in turn, inactivated GSK3α/β [118]. Thus, the increased GSK3 activity stimulated Rac1 and lamellipodia expansion, but the high Rac1 activity led to GSK3 inactivation.

Taken together, the above results strongly suggest that during the mesenchymal mode of cancer cells migration, GSK3 activates Rac1, and hence, its downstream effectors such as WASF2, Arp2/3, and cofilin that leads to induction of lamellipodia formation.

Filopodia are finger-like cell protrusions that also drive cancer cells’ migration during the mesenchymal motility mode. They are formed from linear actin without the branching activity of Arp2/3 [119]. Elongation of these protrusions is associated with Rac1 and Cdc42 activity (for review see [120]). It has been demonstrated that GSK3β promoted filopodia formation and migration of the primary human lung cancer cells [106]. This has been associated with phosphorylation of the long-form collapsin response mediator protein-1 (LCRMP-1) [106], a cancer invasion enhancer [121]. In contrast to the above study, it has also been shown that Cdc42 induced filopodia formation but downregulated GSK3β activity [51,122].

Invadopodia are another actin-rich protrusion important in cancer cells migration and invasion, and their formation also depends on the activity of GSK3. The main role of these protrusions is to penetrate ECM and degrade it with metalloproteinases that allow cancer cells to spread. The association of the actin cytoskeleton with proteins such as Arp2/3 has been shown to be indispensable to the formation of these protrusions [123]. In human glioblastoma cell line U251, the inhibition of GSK3β with AR-A014418 reduced the expression of membrane-type I-matrix metalloproteinase (MT1-MMP). Downregulation of MT1-MMP expression reduced the number of invadopodia [104]. GSK3β activity has been also correlated to the increased expression and secretion of matrix metalloproteinase-2 and 9, which improved the migratory phenotype of synovial sarcoma, fibrosarcoma, osteosarcoma, and pancreatic cancer cell lines [124,125,126].

In the osteosarcoma cell line MG-63, the inhibition of GSK3β with either AR-A014418 or SB216763 also reduced FAK activity [126], which is known to regulate invadopodia formation by sequestration of the invadopodia-inducing Src kinase at focal adhesions [127].

Blebs are cytoplasmic pressure-induced round cellular protrusions required for the induction of amoeboid movement. They do not contain actin at the early stages of their formation. RhoA is the main factor driving the amoeboid mode of movement (for review see [110,128]). In the human gastric cancer cell line SGC-7901, suppression of PI3K/Akt/GSK3β pathway, and hence, activation of GSK3β inhibited Wnt family member 5A (Wnt5A)-induced activation of RhoA [129]. On the other hand, in the thyroid carcinoma cell line FTC-133, Wnt5A reduced cell motility while having no effect on GSK3β phosphorylation status [130]. It has been shown that in the murine RAW264.7 and human HEK 293 cell lines, another Wnt pathway protein, Wnt family member 3A (Wnt3A), induced inhibition of GS3Kβ and activation of RhoA, which stimulated RAW264.7 cells migration [108]. Thus, it might be concluded that active GSK3 basically plays an inhibitory role in the amoeboid, RhoA-dependent cell movement.

It has been shown that a downstream effector of RhoA, Rho-associated protein kinase 1 (ROCK1), phosphorylated and additionally inactivated GS3Kβ [108]. In the human hepatocellular cancer cell line HEPG2, treatment with insulin, an upstream effector downregulating GSK3β, led to the activation of RhoA. In the same study, RhoA/ROCK1 was identified as a non-canonical pathway leading to GSK3β inactivation by insulin [131]. In contrast, it has been shown that in fibroblasts, GSK3β indirectly activated RhoA by phosphorylation and inhibition of RhoGAP, an upstream effector inactivating RhoA [111,132]. This process was regulated by priming by mitogen-activated protein kinases (MAPK) [132].

Taken together, the above results strongly support the hypothesis that GSK3 is positively engaged in the mesenchymal but not an amoeboid form of movement. In the mesenchymal mode, active GSK3 induces Rac1 activity and, subsequently, its downstream effectors, which lead to cell movement due to the formation of lamellipodia, filopodia, and invadopodia. In the amoeboid mode, active GKS3 inactivates RhoA and reduces the movement. Furthermore, during the amoeboid mode of movement, active RhoA stimulates the inhibitory phosphorylation of GSK3. Since different cancer cell lines have specific migration mode preferences [112], the exact role of GSK3 in cancer cells migration may vary depending on the cell type.

### 3.2. Microtubule Plus-End Tracking Proteins in Direct Cell Movement

Polarization of cells is induced by microtubules. Polymerization is an essential event required for directed cell movement [133]. Microtubule-induced polarization requires plus-end tracking proteins (+TIP proteins) such as APC, EB1, and CLASP2, which promote microtubule elongation (for review see [133]).

Numerous studies have demonstrated that APC is bound directly to the plus tip of microtubules, but it could also be attached through kinesin superfamily associated protein 3 (KAP3) [134,135] or through end-binding protein 1 (EB1) [136]. The interaction between APC and microtubules has been shown to be reduced by GSK3β-driven phosphorylation of the C-terminal domain of APC in vitro [62]. Spontaneous mutations of APC in human colorectal cancers led to the formation of a C-terminally truncated form of the protein, which could not be regulated by GSK3 but could still interact (via the N-terminal domain) with MT and stimulate aberrant and untargeted cell migration. The C-terminal domain was responsible for APC binding to MT both directly and via EB1 (for review see [137]). EB1 accumulated at growing microtubule tips and has pivotal roles in the regulation of cell polarity and migration [107]. In primary rat astrocytes, stabilization of the active GSK3β by HYS-32 eliminated EB1 from MT plus ends and reduced cell migration [138]. Simultaneous inhibition of GSK3β by SB415286 attenuated this effect [138]. EB1 is overexpressed in various cancer cells, including glioblastoma, hepatocellular carcinoma, colon, oral, and breast cancers (for review see [139]). In the human non-small lung carcinoma A549 cell line, vincristine (a microtubule-targeting agent) has been shown to induce ROS-mediated decrease of Akt activity and therefore increase the activity of GSK3β [107]. Active GSK3β phosphorylated EB1 that decreased its accumulation at the microtubule plus-end and reduced cell migration. Vincristine reduced also the migration of the U87-MG glioblastoma cancer cell line [107]. Interactions between APC and EB1 have been shown to be promoted by a RhoA downstream effector mDia1 [136]. Upregulation of GSK3β in T lymphocytes from C57Bl/6 mDia1-/- mice resulted in increased APC phosphorylation and impaired cells migration. Active mDia1 downregulated GSK3β [140].

CLASP2 is another GSK3-dependent +TIP protein [141]. CLASP2 has been shown to bind to APC and EB1 and regulate cell polarity and migration [142]. CLASP2 is associated with MT plus ends in cell bodies, and MT lattices in the structure localized behind lamellipodia, called lamella. During MT growth, CLASP2 is also associated with actin by interaction with actin cross-linking factor 7 (ACF7) [142].

In the marsupial kidney epithelial PtK1 cell line, constitutively active GSK3β decreased the association of CLASP2 with MT lattices in lamella [141]. Unexpectedly, also Rac1 (which is activated by GSKβ [103]), has been identified as the upstream effector responsible for promoting the affinity of CLASP2 to MT lattices [141]. In the human keratinocyte HaCaT cell line, constitutively active GSK3β (S9A mutant) decreased the density of MT in lamella and reduced migration [143]. It has been shown to result from phosphorylation of CLASP2 by GSK3β at eight serine residues that altered MT association to CLASP2 [143]. Moreover, these phosphorylation events inhibited the interactions of CLASP2 with MT plus end and with EB1. However, all eight phosphorylated serine residues were required to completely block MT plus-end association with CLASP2 [143]. On the other hand, it has been shown that the physiological activity of GSK3β did not result in phosphorylation of all potential phosphorylation sites on CLASP2, and thus, GSK3β could not entirely disrupt the complex [143]. It has been proposed that a gradient of GSK3β activity might regulate the CLASP2-MT association in the cell [143].

In the human breast SKBr3 carcinoma cell line, receptor tyrosine-protein kinase ErbB2 activity resulted in GSK3β phosphorylation at S9 [142]. ErbB2 is a downstream effector of Memo and RhoA/mDia1 signaling. In this scenario, longer microtubules that infiltrated protrusions were observed [142]. Silencing of APC or CLASP2 negatively affected MT formation without influencing protrusions dynamics [142]. In the presence of physiological activity of GSK3, the overexpression of APC or CLASP2 in SKBr3 cells restored MT localization in protrusions, and in these cells, CLASP2 located to the ruffles and formed comets following growing MT plus tips [142]. However, inhibition of GSK3β with lithium resulted in random and homogenous CLASP2 association to the entire length of microtubules and ruffles. Thus, it has been hypothesized that a gradient of GSK3β activity was needed for the proper CLASP2 associations [142].

ACF7 has been shown to serve as a binding factor between actin and microtubules and to localize to plasma membrane and ruffles as a downstream effector of APC in SKBr3 cells [142]. In hair follicle stem cells (HF-SC), the C-terminal microtubule-binding domain of ACF7 was phosphorylated by GSK3, which led to a reduction in ACF7 affinity in microtubules [144]. In ACF7-deficient cells, directed migration was defective. Although the above-mentioned studies suggested that GSK3β activity was associated with reduced tubulin-dependent migration [138,140,143], it has also been shown that both GSK3β inhibition with lithium and its indirect activation with wortmannin impaired cell migration [144]. This study has suggested that the spatiotemporal regulation of GSK3β activity was necessary for cells migration [144].

Overall, several lines of evidence demonstrate a negative role for GSK3β activity in cell polarity and directed movement, as a result of +TIPs phosphorylation [138,140,143], although GSK3β activity does not simply inhibit cells migration. The experiments that demonstrated reduced microtubule-related movement were mostly performed with the use of cells expressing constitutively active GSK3β, or in the presence of blockers of physiological inhibitors of GSK3β [138,140,143]. On the other hand, studies that centered on actin and Rac1 demonstrated the stimulatory role of GSK3β activity on cell migration [104,105,115,116,117]. These studies were performed only in cells where the activity of the kinase was downregulated by inhibitors or mRNA silencing [104,105,115,116,117]. When both overactivation and inhibition of GSK3β were analyzed, it was demonstrated that reduced and increased activity of GSK3β mitigated migration [144].

To summarize, the role(s) of GSK3β in the migration of cells seems to be opposite depending on whether it is studied in the context of tubulin or actin. It appears that both downregulation and overactivity/upregulation of the kinase reduce cells migration. Evidently, the optimal activity of the kinase and its spatiotemporal distribution are required to maintain the migratory potential of the cells.

### 3.3. Focal Adhesion

Focal adhesions are structures where the actin cytoskeleton is connected to integrins, which are responsible for cell attachment to ECM. This allows force transfer during cell migration. Actin fibers are connected to integrins through a protein complex comprising of FAK, vinculin, paxillin, and other proteins [145]. FA are formed in protrusions of migrating cells and are important during both cell formation and retraction of protrusions (for review see [146]). FAK is the central mediator of FA turnover, and its activity results in the formation of the FAK-Src complex that phosphorylates paxillin [147]. This causes FA disassembly on the rear of migrating cells [147].

In Rat2 fibroblasts, synthetic triterpenoids treatment inhibiting GSK3 resulted in enlarged FA and thus reduced motility [114]. It has been shown that inhibition of GSK3 by lithium or SB216763, or its silencing, inhibited HeLa cells’ mobility [148]. Active GSK3α/β is associated with the human homolog of Drosophila prune protein (h-prune), which is colocalized with FA proteins paxillin and vinculin [148]. SB216763 treatment resulted in the formation of larger FA, impaired FA turnover, and also the limited formation of new FA in lamellipodia [148]. It has been demonstrated that the GSK3-h-prune complex stimulated FAK autophosphorylation at Y397 and its activation [148]. Silencing or inhibition of GSK3β with NP309 or lithium reduced movement of human melanoma cell lines (WM793, 1205Lu, and WM9), and FA were larger when FAK Y397 was not phosphorylated [149]. Migration of the human osteosarcoma cell line (MG-63) has been shown to be reduced by GSK3 inhibitors AR-A014418 and SB216763, and when FAK Y397 phosphorylation was decreased [126]. Autophosphorylation of FAK and cells migration was also reduced in three pancreatic cancer cell lines (MIA PaCa-2, PANC-1, and BxPC-3) [105] and two human glioblastoma cell lines (T98G and U87) [104]) after AR-A014418 treatment [104,105]. On the other hand, in rat fibroblasts, GSK3β interacted with and phosphorylated FAK at S722 [10]. Since lithium blocked FAK phosphorylation at S722 and also increased cells motility, it has been hypothesized that the phosphorylation status of S722 was important for cell spreading [10].

Additionally, paxillin is a target of direct phosphorylation by GSK3β [150]. In RAW 264.7 mouse macrophages, paxillin phosphorylation at S126 was attenuated after lithium, GSK-3 inhibitor IX or GSK-3 inhibitor I treatment [150]. Priming phosphorylation at S130 by ERK was necessary for the GSK3β-mediated phosphorylation [150]. The stimulation of cells movement by GSK3-mediated phosphorylation of paxillin was also observed in a conditionally immortalized podocytes cell line, where inhibition of GSK3 by lithium reduced turnover of FA and inhibited podocyte migration [151]. Additionally, it was demonstrated that Rac1, whose activity depends on GSK3, stimulated PAK-dependent paxillin phosphorylation at S273 in the Chinese hamster ovary CHO-K1 cell line, which led to increased FA turnover and increased motility [152].

FA disassembly is also dependent on MT as they transport autophagosomes to FA [153]. In vitro experiments with murine skin samples and nocodazole, an MT depolymerizing agent, have demonstrated a reduced FA turnover [154]. The same effect was observed in keratinocytes from mice with ablated expression of ACF7–a protein that connects actin and MT cytoskeleton, in the skin [154]. In primary mouse keratinocytes, ACF7 activated by FAK-Src guided growing microtubules toward FA [155]. The microtubule +TIP proteins APC [153] and CLASP2 [143] were involved in FA turnover and migration of the human breast carcinoma MDA-MB-231 cell line [153] and the human keratinocyte HaCaT cell line [143].

Although it has been shown that GSK3β-driven FAK activation is indispensable for FA turnover, several studies have demonstrated that GSK3β inhibited proteins such as ACF7, APC, and CLASP2 [140,143,144], which were engaged in FA disassembly and hence, FA turnover [143,153,154]. However, it has also been demonstrated that active FAK could inhibit, via stimulation of PI3K/Akt, GSK3β activity [156], and thus, a negative feedback interaction between these two kinases may be expected.

**Figure 3:**
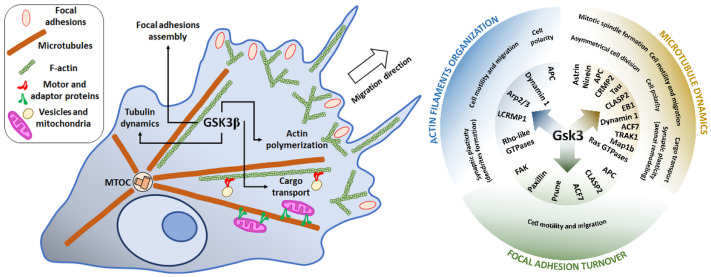
GSK3β is involved in the regulation of cell motility and migration and also the transport of cargo (e.g., mitochondria that supply energy for the processes) along the cytoskeleton. The activity of the kinase is necessary for proper polarization of the cell and formation of protrusions and thus for regulation of migratory potential of the cell. The main protein targets of GSK3 described in this paper, and the cytoskeleton-related processes in which they participate are presented in the right panel of the figure. For more detailed information see the text and Supplementary Tables S1 and S2.

These cell migration-related processes and the main protein targets of GSK3 are briefly summarized in Figure 3. More detailed information on the targets and GSK3-regulating kinases/pathways are presented in Supplementary Tables S1 and S2.

## 4. GSK3β–Cytoskeleton Interplay in Mitochondria Trafficking

Mitochondria are the powerhouses of the cells by producing a major supply of cellular ATP. Mitochondria act also as metabolic centers of cells by regulating numerous processes, e.g., fatty acid oxidation, glutamate, and urea metabolism. Additionally, mitochondria are an important Ca^2+^ reservoir, have a buffering capacity, and control apoptosis [157,158]. Malfunctioning mitochondria are the causes of a number of diseases affecting especially tissues with a high metabolic rate, such as the brain, liver, heart, muscles, and glands [159]. To properly perform their functions, mitochondria must dynamically change the structure of their network and travel to specific cellular regions. Mitochondrial fusion and fission facilitate repair of the organelles, or (when irreversibly damaged) their removal by autophagy (mitophagy) [160]. Energy demands of individual regions of the cell are not the same, especially in polarized cells such as neurons [158]. This demand is met by the active movement of mitochondria along microtubules [161] in two directions: anterogradely (to microtubule plus end, mostly toward the cell membrane) and retrogradely (to microtubule minus end, mostly toward the nucleus) [162]. The organelles are transported by two motor proteins families, which use GTP to slide along microtubules. Anterograde movement of mitochondria is driven by kinesins, retrograde by dyneins. Mitochondrial outer membrane and motor protein are linked by large protein complexes, which regulate mitochondrial movement velocity and pausing-starting events [163]. Alteration of mobility might result in numerous neuropathological conditions such as Alzheimer’s disease, Huntington’s disease, psychotic disorders, and dementia [164]. The mitochondrial movement is also at the root of tumor plasticity [165]. The multifunctional GSK3 participates in the pathogenesis of these diseases also by impacting mitochondrial mobility.

Proper mitochondrial trafficking is crucial to neuronal synapse formation and signal transduction. In the case of dysfunction of the organelles in synapses, they are transported along microtubules to the cell body, where they are repaired or removed by mitophagy if the repair is unprofitable. Additionally, in the cell body, mitochondria biogenesis occurs. New mitochondria are then transported to the synapse to support energetically and metabolically neurotransmission [166].

It has been shown that in AD models, mitochondria have decreased mobility in comparison to healthy cells [158], which might result in synaptic failure [167]. There are two explanations for this reduction in trafficking. Firstly, it has been observed that amyloid-beta (Aβ) reduced the expression of KIF5A, a key isoform of kinesin-1, critical for neuronal mitochondrial transport [167].

**Figure 4:**
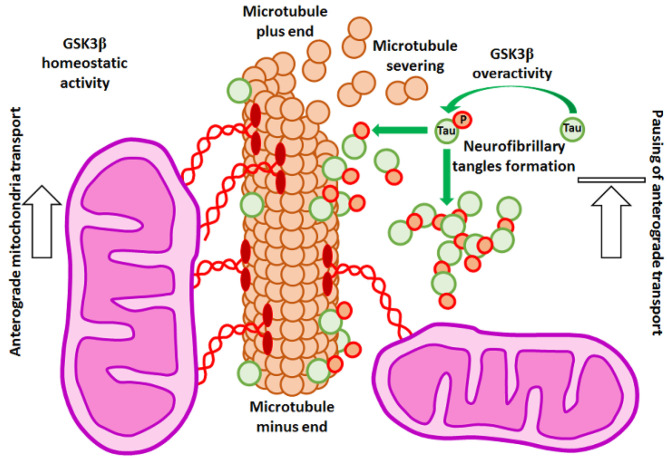
GSK3β influences anterograde transport of organelles in neurons. Overactivity of the kinase results in hyperphosphorylation of Tau protein that leads to the formation of neurofibrillary tangles and facilitates the severing of microtubules. This, in turn, results in pausing of the anterograde transport of mitochondria and neurotransmitter-carrying vesicles.

Secondly, it has been suggested that non-motor microtubule-associated proteins such as Tau might be responsible for this phenomenon, and it is generally accepted that Tau overexpression stops mitochondria. It has been shown that Tau overexpressed during the onset of neurodegeneration accumulated in neurons and acted as a “roadblock” for motor proteins, reducing their access to microtubules [168]. In response to this, hyperphosphorylation of Tau ensued. In consequence, Tau created insoluble neurofibrillary tangles that dissociated from microtubules, which became more prone to the actions of depolymerizing agents [168] and, in turn, resulted in mitochondria pausing (Figure 4). It has been also suggested that the soluble form of Tau impaired mobility of the organelles, but the exact mechanism of this was not fully explained [169].

GSK3 is a Tau kinase overactivated in AD neurons [97]. Studies with a cell line model of differentiated neurons have shown that GSK3β overexpression resulted in Tau hyperphosphorylation and mitochondrial transport alteration. It caused mitochondrial pausing and impaired trafficking preferentially in the anterograde direction [170]. Therefore, inhibition of GSK3β by LiCl or more specific inhibitors (such as SB216763; for the convenience of the reader, information about the properties of GSK3 inhibitors used in the experiments cited in this article has been summarized in Table 1) is used in AD treatment [171].

It has been shown that either LiCl or SB216763 treatment resulted in an increased number of motile mitochondria and elevated mitochondrial velocity. It has been proposed that the restored number of fully functional mitochondria in synapses could prevent or even abolish some symptoms of AD [172]. On the other hand, it has been shown that phosphorylation of Tau by GSK3β was crucial to maintain mitochondrial trafficking in neurons. In mouse hippocampal neurons, GSK3β activity increased the number of motile mitochondria in a Tau-dependent manner [173]. These results emphasize the need to maintain the proper level of GSK3 activity to maintain normal functions of cells.

Dysregulation of GSK3 is also involved in other neurological disorders. GSK3β inhibitory phosphorylation has been found to be reduced in lymphocytes of people suffering from schizophrenia [174]. Therefore, GSK3β inhibitors such as SB216763 and 1-azakenpaullone were proposed as antipsychotic drugs [175]. Their action may be based on an influence on interactions between GSK3, DISC1, and TRAK1 proteins. It has been shown that TRAK1 acted as the link between motor proteins (dynein and kinesin) and tubulin, and DISC1 and GSK3β together regulated TRAK1 activity what resulted in the anterograde movement of mitochondria [176]. This interaction between the three proteins may be crucial in schizophrenia etiology since DISC1 gene disruption is widely known as a predictor of schizophrenia development [177].

GSK3 could be also a metastasis regulating factor. In the general tumor population, there is a subpopulation of cancer stem cells (CSCs) that resist oncological treatments and contribute to relapse. CSCs are also accused of contributing to metastatic events. Migrating CSCs consume a lot of energy derived by mitochondria, which must be repositioned to the lamellipodia [178]. Thus, proper mitochondrial dynamics and trafficking are essential for metastasis. During migration and invasion, the organelles actively travel to leading-edge lamellipodia, where they increase local ATP concentration [179].

The PI3K/AKT pathway is overactive in many cancers, promoting a high proliferation rate of cancer cells. Therefore, PI3K/AKT inhibitors such as idelalisib are used as anti-cancer drugs during chemotherapy [180]. However, PI3K/AKT inhibition is related to enhanced mitochondrial motility and their relocalization into the lamellipodia of cancer cells, resulting in increased migratory potential of the cells [181]. Since activation of the PI3K/AKT pathway is responsible for inhibitory phosphorylation of GSK3 (on Ser9), reduction of this pathway activity should enhance the GSK3-induced mitochondrial repositioning into lamellipodia and thus escalate the risk of metastasis.

This once again shows that we still need a more complete understanding of the complex roles that signaling pathways and their components play in the cell.

Mitochondria, together with the endoplasmic reticulum, are fundamental also for the maintenance of calcium homeostasis because they can sequester and release the ion regulating its local and global concentration in the cell. Thus, proper intracellular distribution of mitochondria is essential in cells that experience frequent and large fluctuations in the concentration of Ca^2+^, such as cardiac myocytes. In turn, it has been shown that in cardiac myoblasts, physiological oscillations of Ca^2+^ concentrations inhibited mitochondrial trafficking along microtubules to enhance buffering of the ion in the compartments where it was most needed. Moreover, increased Ca^2+^ buffering by mitochondria could also stimulate mitochondrial ATP production ensuring that region-specific energy requirements are met [182].

In cardiomyocytes of adult animals, mitochondria are static and their mobility is limited. The population of the organelles can be divided into three separate groups: subsarcolemmal mitochondria (SSM), intermyofibrillar mitochondria (IMF), or perinuclear mitochondria (PNM). Only PNM have high mobility and are probably involved in transcription and translation. IMF are in continuous interaction with the sarcoplasmic reticulum to allow optimal buffering energy transfer (see in [183]). Therefore, disturbances in the intracellular distribution of mitochondria might result in impairment of heart contractile function. It has been observed in a murine model of Duchenne dystrophy [184] and in aged cardiomyocytes [185]. In neonate cardiomyocytes, mitochondria are much more mobile and distributed along microtubules. Thus, taking into account that GSK3 modulates the stability and dynamics of microtubules, it should also affect the mobility of organelles. Additionally, using the H9c2 cardiac myoblasts cell line, it has been shown that movement of the mitochondria was facilitated by their anchorage to motor proteins through a subfamily of Ras GTPases, Miro 1 and Miro 2 proteins. Miros acted as Ca^2+^ sensors and facilitated mitochondria trafficking along microtubules, but they were also responsible for the arrest of the organelles in the proper place [186]. TRAK1 is a key protein that links molecular motors to Miro. As it has been described above, GSK3β bound to TRAK1, and together with other proteins, promoted mitochondrial trafficking. Loosening of the interactions by GSK3 inhibitors impaired the trafficking [176].

Quite recently, it has been shown that APC was one of the proteins responsible for interactions of mitochondria with Miro/Milton–motor protein complex, and it was needed for initiation of mitochondrial movement [187]. APC is a target of GSK3 and it stabilizes microtubules when GSK3 is inhibited (see above). However, it has been suggested that APC acted as a positive regulator of the Miro/Milton/KIF5 complex independently of its influence on the cytoskeleton [187] but, supposedly, not independently on GSK3 activity. Since mitochondrial trafficking in cardiac myoblasts is driven by the same proteins as in neuronal cells, a regulatory role of GSK3 in the heart can also be expected.

It should be noted, however, that there are not many publications on the trafficking of mitochondria in the heart because, in contrast to myoblasts and neonatal cardiomyocytes, in adult cardiomyocytes, mitochondrial fusion–fusion processes seem to play a more important role than the trafficking in the normal function of the organ. GSK3 can also take part in the regulation of these processes, but this is a topic for another research.

## 5. Conclusions

For many years, scientists and clinicians have focused their attention on the biochemical mechanisms of GSK3 and its involvement in various diseases. Additionally, for many years, this kinase has been indicated as a potential target of various therapies, and thus, efforts have been concentrated on exploring the possibility of using GSK3 inhibitors as potential drugs. Unfortunately, stunning success has not been frequently observed. In the way of achieving success stands probably the very reason why GSK3 is such an interesting object of research: multifunctionality of the kinase and complexity of functional cross-talks between GSK3 and its up- and downstream effectors. Global inhibition or overexpression of GSK3 in the cell rarely produces the desired results, often leading instead to serious side effects. Thus, although numerous GSK3 inhibitors have been evaluated, they relatively rarely reach Phase-2 clinical trials. The conclusions drawn in the experimental papers reviewed in our paper suggest that also in the case of the cytoskeleton-related cellular roles of GSK3, e.g., regulation of division, migratory potential, and the polarity of the cell, and maintaining transport of cargo along the cytoskeleton, a precise balance between activation and inhibition of the kinase and its proper spatiotemporal distribution is required. Thus, focusing on finding methods that enable only local alteration of activity/concentration of this protein, or targeting a specific downstream target or an upstream regulator of GSK3 might be a more promising approach than changing the total cellular activity/concentration of the kinase. Therefore, a comprehensive description of the cellular interaction networks of GSK3 might be crucial to identify novel protein targets with the highest therapeutic potential for the treatment of GSK3-related diseases.

## Figures and Tables

**Table 1:** Description of GSK3 inhibitors used in experiments reviewed in this paper.

Inhibitor	Structure	Formula	Molecular Weight	IC50	Soluble in	Selectivity
Lithium (LiCl)	Li–Cl	LiCl	42.39	1.0 mM	DMSO, ethanol, water, and other solvents	Also inhibits protein kinase A, casein kinase II, MEK/ERK, ERK1, SAPK, Raf, and Trk
SB216763	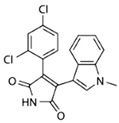	C_19_H_12_N_2_O_2_Cl_2_	371.22	34.3 nM	DMSO	high
SB415286	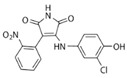	C_16_H_10_ClN_3_O_5_	359.72	78 nM	DMSO ethanol	high
I3′M	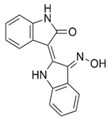	C_16_H_11_N_3_O_2_	277.28	22nM	DMSO	Also inhibits CDK5/p25, CDK1/cyclin B and 5-Lipoxygenase
Kenpaullone	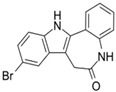	C_16_H_11_BrN_2_O	327.18	23 nM	DMSO	Also inhibits CDK2/cyclin A, CDK2/cyclin E, and CDK5/p25
1-Azakenpaullone	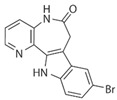	C_15_H_10_BrN_3_O	328.16	18 nM	DMSO	Also inhibits CDK1/cyclin B and CDK5/p25
GSK 3 Inhibitor IX	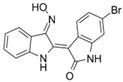	C_16_H_10_BrN_3_O_2_	356.17	5 nM	DMSO	Also inhibits CDK1 and CDK5
AR-A0114418	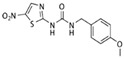	C_12_H_12_N_4_O_4_S	308.31	104 nM	DMSO	high

## Data Availability

Not applicable.

## Supplementary Materials

The following are available online at https://www.mdpi.com/article/10.3390/cells10082092/s1, Table S1: Main (direct and indirect) GSK3 targets mentioned in the present paper, their cellular roles, major effects of the GSK3-target protein interactions, together with the experimental model and methods of detection used in the reviewed papers, Table S2: Main upstream proteins/pathways regulating GSK3β activity mentioned in the present paper, cellular processes they stimulate, and their influence on the activity of GSK3β, together with the experimental model and methods of detection used in the reviewed papers.

## References

[1] Embi N., Rylatt D.B., Cohen P. (1980). Glycogen synthase kinase-3 from rabbit skeletal muscle. Separation from cyclic-AMP-dependent protein kinase and phosphorylase kinase. Eur. J. Biochem..

[2] Woodgett J.R. (1990). Molecular cloning and expression of glycogen synthase kinase-3/factor A. EMBO J..

[3] Kaidanovich-Beilin O., Lipina T.V., Takao K., van Eede M., Hattori S., Laliberté C., Khan M., Okamoto K., Chambers J.W., Fletcher P.J. (2009). Abnormalities in brain structure and behavior in GSK-3alpha mutant mice. Mol. Brain..

[4] Soutar M.P., Kim W.Y., Williamson R., Peggie M., Hastie C.J., McLauchlan H., Snider W.D., Gordon-Weeks P.R., Sutherland C. (2010). Evidence that glycogen synthase kinase-3 isoforms have distinct substrate preference in the brain. J. Neurochem..

[5] McCubrey J.A., Steelman L.S., Bertrand F.E., Davis N.M., Sokolosky M., Abrams S.L., Montalto G., D’Assoro A.B., Libra M., Nicoletti F. (2014). GSK-3 as potential target for therapeutic intervention in cancer. Oncotarget.

[6] Duda P., Wiśniewski J., Wójtowicz T., Wójcicka O., Jaśkiewicz M., Drulis-Fajdasz D., Rakus D., McCubrey J.A., Gizak A. (2018). Targeting GSK3 signaling as a potential therapy of neurodegenerative diseases and aging. Expert Opin. Ther. Targets.

[7] Bhat R.V., Andersson U., Andersson S., Knerr L., Bauer U., Sundgren-Andersson A.K. (2018). The Conundrum of GSK3 Inhibitors: Is it the Dawn of a New Beginning?. J. Alzheimers Dis..

[8] Sun T., Rodriguez M., Kim L. (2009). Glycogen synthase kinase 3 in the world of cell migration. Dev. Growth Differ..

[9] Xu W., Ge Y., Liu Z., Gong R. (2015). Glycogen synthase kinase 3β orchestrates microtubule remodeling in compensatory glomerular adaptation to podocyte depletion. J. Biol. Chem..

[10] Bianchi M., De Lucchini S., Marin O., Turner D.L., Hanks S.K., Villa-Moruzzi E. (2005). Regulation of FAK Ser-722 phosphorylation and kinase activity by GSK3 and PP1 during cell spreading and migration. Biochem. J..

[11] Luo J. (2012). The Role of GSK3beta in the Development of the Central Nervous System. Front. Biol..

[12] Leroy K., Brion J.P. (1999). Developmental Expression and Localization of Glycogen Synthase Kinase-3β in Rat Brain. J. Chem. Neuroanat..

[13] Takahashi M., Tomizawa K., Ishiguro K. (2000). Distribution of Tau Protein Kinase I/Glycogen Synthase Kinase-3β, Phosphatases 2A and 2B, and Phosphorylated Tau in the Developing Rat Brain. Brain Res..

[14] Drulis-Fajdasz D., Rakus D., Wiśniewski J.R., McCubrey J.A., Gizak A. (2018). Systematic Analysis of GSK-3 Signaling Pathways in Aging of Cerebral Tissue. Adv. Biol. Regul..

[15] Lee S.J., Chung Y.H., Joo K.M., Lim H.C., Jeon G.S., Kim D., Lee W.B., Kim Y.S., Cha C.I. (2006). Age-Related Changes in Glycogen Synthase Kinase 3β (GSK3β) Immunoreactivity in the Central Nervous System of Rats. Neurosci. Lett..

[16] Spittaels K., Van Den Haute C., Van Dorpe J., Geerts H., Mercken M., Bruynseels K., Lasrado R., Vandezande K., Laenen I., Boon T. (2000). Glycogen Synthase Kinase-3β Phosphorylates Protein Tau and Rescues the Axonopathy in the Central Nervous System of Human Four-Repeat Tau Transgenic Mice. J. Biol. Chem..

[17] Spittaels K., Van den Haute C., Van Dorpe J., Terwel D., Vandezande K., Lasrado R., Bruynseels K., Irizarry M., Verhoye M., Van Lint J. (2002). Neonatal Neuronal Overexpression of Glycogen Synthase Kinase-3β Reduces Brain Size in Transgenic Mice. Neuroscience.

[18] Cui H., Meng Y., Bulleit R.F. (1998). Inhibition of Glycogen Synthase Kinase 3β Activity Regulates Proliferation of Cultured Cerebellar Granule Cells. Dev. Brain Res..

[19] Boku S., Nakagawa S., Masuda T., Nishikawa H., Kato A., Kitaichi Y., Inoue T., Koyama T. (2009). Glucocorticoids and Lithium Reciprocally Regulate the Proliferation of Adult Dentate Gyrus-Derived Neural Precursor Cells through GSK-3β and β-Catenin/TCF Pathway. Neuropsychopharmacology.

[20] Maurer M.H., Brömme J.O., Feldmann R.E., Järve A., Sabouri F., Bürgers H.F., Schelshorn D.W., Krüger C., Schneider A., Kuschinsky W. (2007). Glycogen Synthase Kinase 3β (GSK3β) Regulates Differentiation and Proliferation in Neural Stem Cells from the Rat Subventricular Zone. J. Proteome Res..

[21] Yeste-Velasco M., Folch J., Trullàs R., Abad M.A., Enguita M., Pallàs M., Camins A. (2007). Glycogen Synthase Kinase-3 Is Involved in the Regulation of the Cell Cycle in Cerebellar Granule Cells. Neuropharmacology.

[22] Cheng T.S., Hsiao Y.L., Lin C.C., Yu C.T.R., Hsu C.M., Chang M.S., Lee C.I., Huang C.Y.F., Howng S.L., Hong Y.R. (2008). Glycogen Synthase Kinase 3β Interacts with and Phosphorylates the Spindle-Associated Protein Astrin. J. Biol. Chem..

[23] Mack G.J., Compton D.A. (2001). Analysis of Mitotic Microtubule-Associated Proteins Using Mass Spectrometry Identifies Astrin, a Spindle-Associated Protein. Proc. Natl. Acad. Sci. USA.

[24] Tighe A., Ray-Sinha A., Staples O.D., Taylor S.S. (2007). GSK-3 Inhibitors Induce Chromosome Instability. BMC Cell Biol..

[25] Hong Y.R., Chen C.H., Chang J.H., Wang S.K., Sy W.D., Chou C.K., Howng S.L. (2000). Cloning and Characterization of a Novel Human Ninein Protein That Interacts with the Glycogen Synthase Kinase 3β. Biochim. Biophys. Acta-Gene Struct. Expr..

[26] Howng S.L., Hsu H.C., Cheng T.S., Lee Y.L., Chang L.K., Lu P.J., Hong Y.R. (2004). A Novel Ninein-Interaction Protein, CGI-99, Blocks Ninein Phosphorylation by GSK3β and Is Highly Expressed in Brain Tumors. FEBS Lett..

[27] Ma C., Wang J., Gao Y., Gao T.W., Chen G., Bower K.A., Odetallah M., Ding M., Ke Z., Luo J. (2007). The Role of Glycogen Synthase Kinase 3β in the Transformation of Epidermal Cells. Cancer Res..

[28] Luo J. (2009). Glycogen Synthase Kinase 3β (GSK3β) in Tumorigenesis and Cancer Chemotherapy. Cancer Lett..

[29] Malliri A., Symons M., Hennigan R.F., Hurlstone A.F.L., Lamb R.F., Wheeler T., Ozanne B.W. (1998). The Transcription Factor AP-1 Is Required for EGF-Induced Activation of Rho-like GTPases, Cytoskeletal Rearrangements, Motility, and in Vitro Invasion of A431 Cells. J. Cell Biol..

[30] Spiering D., Hodgson L. (2011). Dynamics of the Rho-Family Small GTPases in Actin Regulation and Motility. Cell Adhes. Migr..

[31] Govek E.E., Newey S.E., Van Aelst L. (2005). The Role of the Rho GTPases in Neuronal Development. Genes Dev..

[32] Kalpachidou T., Spiecker L., Kress M., Quarta S. (2019). Rho GTPases in the Physiology and Pathophysiology of Peripheral Sensory Neurons. Cells.

[33] Sui Z., Sniderhan L.F., Fan S., Kazmierczak K., Reisinger E., Kovács A.D., Potash M.J., Dewhurst S., Gelbard H.A., Maggirwar S.B. (2006). Human Immunodeficiency Virus-Encoded Tat Activates Glycogen Synthase Kinase-3β to Antagonize Nuclear Factor-ΚB Survival Pathway in Neurons. Eur. J. Neurosci..

[34] Kim S.D., Yang S.I., Kim H.C., Shin C.Y., Ko K.H. (2007). Inhibition of GSK-3β Mediates Expression of MMP-9 through ERK1/2 Activation and Translocation of NF-ΚB in Rat Primary Astrocyte. Brain Res..

[35] Shimizu T., Kagawa T., Inoue T., Nonaka A., Takada S., Aburatani H., Taga T. (2008). Stabilized β-Catenin Functions through TCF/LEF Proteins and the Notch/RBP-Jκ Complex to Promote Proliferation and Suppress Differentiation of Neural Precursor Cells. Mol. Cell. Biol..

[36] Minde D.P., Anvarian Z., Rüdiger S.G.D., Maurice M.M. (2011). Messing up Disorder: How Do Missense Mutations in the Tumor Suppressor Protein APC Lead to Cancer?. Mol. Cancer.

[37] Minde D.P., Radli M., Forneris F., Maurice M.M., Rüdiger S.G.D. (2013). Large Extent of Disorder in Adenomatous Polyposis Coli Offers a Strategy to Guard Wnt Signalling against Point Mutations. PLoS ONE.

[38] MacDonald B.T., Tamai K., He X. (2009). Wnt/β-Catenin Signaling: Components, Mechanisms, and Diseases. Dev. Cell.

[39] Rao T.P., Kühl M. (2010). An Updated Overview on Wnt Signaling Pathways: A Prelude for More. Circ. Res..

[40] Krylova O., Messenger M.J., Salinas P.C. (2000). Dishevelled-1 Regulates Microtubule Stability: A New Function Mediated by Glycogen Synthase Kinase-3β. J. Cell Biol..

[41] Staal F.J.T., Clevers H. (2000). Tcf/Lef Transcription Factors during T-Cell Development: Unique and Overlapping Functions. Hematol. J..

[42] Bhat R.V., Shanley J., Correll M.P., Fieles W.E., Keith R.A., Scott C.W., Lee C.M. (2000). Regulation and Localization of Tyrosine216 Phosphorylation of Glycogen Synthase Kinase-3β in Cellular and Animal Models of Neuronal Degeneration. Proc. Natl. Acad. Sci. USA.

[43] Ciani L., Salinas P.C. (2007). C-Jun N-Terminal Kinase (JNK) Cooperates with GSK3β to Regulate Dishevelled-Mediated Microtubule Stability. BMC Cell Biol..

[44] Barth A.I.M., Caro-Gonzalez H.Y., Nelson W.J. (2008). Role of Adenomatous Polyposis Coli (APC) and Microtubules in Directional Cell Migration and Neuronal Polarization. Semin. Cell Dev. Biol..

[45] Meixner A., Haverkamp S., Wässle H., Führer S., Thalhammer J., Kropf N., Bittner R.E., Lassmann H., Wiche G., Propst F. (2000). MAP1B Is Required for Axon Guidance and Is Involved in the Development of the Central and Peripheral Nervous System. J. Cell Biol..

[46] González-Billault C., Del Río J.A., Ureña J.M., Jiménez-Mateos E.M., Barallobre M.J., Pascual M., Pujadas L., Simó S., La Torre A., Gavin R. (2005). A Role of MAP1B in Reelin-Dependent Neuronal Migration. Cereb. Cortex.

[47] Kuo T.Y., Hong C.J., Hsueh Y.P. (2009). Bcl11A/CTIP1 Regulates Expression of DCC and MAP1b in Control of Axon Branching and Dendrite Outgrowth. Mol. Cell. Neurosci..

[48] Bouquet C., Soares S., Von Boxberg Y., Ravaille-Veron M., Propst F., Nothias F. (2004). Microtubule-Associate Protein 1B Controls Directionality of Growth Cone Migration and Axonal Branching in Regeneration of Adult Dorsal Root Ganglia Neurons. J. Neurosci..

[49] Fabry B., Klemm A.H., Kienle S., Schäffer T.E., Goldmann W.H. (2011). Focal Adhesion Kinase Stabilizes the Cytoskeleton. Biophys. J..

[50] Burridge K., Turner C.E., Romer L.H. (1992). Tyrosine Phosphorylation of Paxillin and Pp125FAK Accompanies Cell Adhesion to Extracellular Matrix: A Role in Cytoskeletal Assembly. J. Cell Biol..

[51] Etienne-Manneville S., Hall A. (2003). Cdc42 Regulates GSK-3β and Adenomatous Polyposis Coli to Control Cell Polarity. Nature.

[52] Brozzi F., Arcuri C., Giambanco I., Donato R. (2009). S100B Protein Regulates Astrocyte Shape and Migration via Interaction with Src Kinase: Implications for Astrocyte Development, Activation, and Tumor Growth. J. Biol. Chem..

[53] Shi S.H., Cheng T., Jan L.Y., Jan Y.N. (2004). APC and GSK-3β Are Involved in MPar3 Targeting to the Nascent Axon and Establishment of Neuronal Polarity. Curr. Biol..

[54] Shelly M., Cancedda L., Heilshorn S., Sumbre G., Poo M. (2007). ming LKB1/STRAD Promotes Axon Initiation During Neuronal Polarization. Cell.

[55] Choi Y.J., Di Nardo A., Kramvis I., Meikle L., Kwiatkowski D.J., Sahin M., He X. (2008). Tuberous Sclerosis Complex Proteins Control Axon Formation. Genes Dev..

[56] Li Y.H., Werner H., Püschel A.W. (2008). Rheb and MTOR Regulate Neuronal Polarity through Rap1B. J. Biol. Chem..

[57] Fan Z., Li C., Qin C., Xie L., Wang X., Gao Z., Qiangbacuozhen, Wang T., Yu L., Liu H. (2014). Role of the PI3K/AKT Pathway in Modulating Cytoskeleton Rearrangements and Phenotype Switching in Rat Pulmonary Arterial Vascular Smooth Muscle Cells. DNA Cell Biol..

[58] Jiang H., Guo W., Liang X., Rao Y. (2005). Both the Establishment and the Maintenance of Neuronal Polarity Require Active Mechanisms: Critical Roles of GSK-3β and Its Upstream Regulators. Cell.

[59] Yoshimura T., Kawano Y., Arimura N., Kawabata S., Kikuchi A., Kaibuchi K. (2005). GSK-3β Regulates Phosphorylation of CRMP-2 and Neuronal Polarity. Cell.

[60] Cole A.R., Knebel A., Morrice N.A., Robertson L.A., Irving A.J., Connolly C.N., Sutherland C. (2004). GSK-3 Phosphorylation of the Alzheimer Epitope within Collapsin Response Mediator Proteins Regulates Axon Elongation in Primary Neurons. J. Biol. Chem..

[61] Kim W.Y., Zhou F.Q., Zhou J., Yokota Y., Wang Y.M., Yoshimura T., Kaibuchi K., Woodgett J.R.R., Anton E.S., Snider W.D.D. (2006). Essential Roles for GSK-3s and GSK-3-Primed Substrates in Neurotrophin-Induced and Hippocampal Axon Growth. Neuron.

[62] Zumbrunn J., Kinoshita K., Hyman A.A., Näthke I.S. (2001). Binding of the Adenomatous Polyposis Coli Protein to Microtubules Increases Microtubule Stability and Is Regulated by GSK3β Phosphorylation. Curr. Biol..

[63] Zhou F.Q., Zhou J., Dedhar S., Wu Y.H., Snider W.D. (2004). NGF-Induced Axon Growth Is Mediated by Localized Inactivation of GSK-3β and Functions of the Microtubule plus End Binding Protein APC. Neuron.

[64] Zhao Z., Wang Z., Gu Y., Feil R., Hofmann F., Ma L. (2009). Regulate Axon Branching by the Cyclic GMP Pathway via Inhibition of Glycogen Synthase Kinase 3 in Dorsal Root Ganglion Sensory Neurons. J. Neurosci..

[65] Zhao Z., Ma L. (2009). Regulation of Axonal Development by Natriuretic Peptide Hormones. Proc. Natl. Acad. Sci. USA.

[66] Krylova O., Herreros J., Cleverley K.E., Ehler E., Henriquez J.P., Hughes S.M., Salinas P.C. (2002). WNT-3, Expressed by Motoneurons, Regulates Terminal Arborization of Neurotrophin-3-Responsive Spinal Sensory Neurons. Neuron.

[67] Bilimoria P.M., De La Torre-Ubieta L., Ikeuchi Y., Becker E.B.E., Reiner O., Bonni A. (2010). A JIP3-Regulated GSK3β/DCX Signaling Pathway Restricts Axon Branching. J. Neurosci..

[68] Kwon C.H., Luikart B.W., Powell C.M., Zhou J., Matheny S.A., Zhang W., Li Y., Baker S.J., Parada L.F. (2006). Pten Regulates Neuronal Arborization and Social Interaction in Mice. Neuron.

[69] Drinjakovic J., Jung H., Campbell D.S., Strochlic L., Dwivedy A., Holt C.E. (2010). E3 Ligase Nedd4 Promotes Axon Branching by Downregulating PTEN. Neuron.

[70] Dent E.W., Kalil K. (2001). Axon Branching Requires Interactions between Dynamic Microtubules and Actin Filaments. J. Neurosci..

[71] Gerrow K., Triller A. (2010). Synaptic Stability and Plasticity in a Floating World. Curr. Opin. Neurobiol..

[72] Gaiarsa J.L., Caillard O., Ben-Ari Y. (2002). Long-Term Plasticity at GABAergic and Glycinergic Synapses: Mechanisms and Functional Significance. Trends Neurosci..

[73] Sorra K.E., Harris K.M. (2000). Overview on the Structure, Composition, Function, Development, and Plasticity of Hippocampal Dendritic Spines. Hippocampus.

[74] Lei W., Omotade O.F., Myers K.R., Zheng J.Q. (2016). Actin Cytoskeleton in Dendritic Spine Development and Plasticity. Curr. Opin. Neurobiol..

[75] Hlushchenko I., Koskinen M., Hotulainen P. (2016). Dendritic Spine Actin Dynamics in Neuronal Maturation and Synaptic Plasticity. Cytoskeleton.

[76] Koivisto L., Häkkinen L., Matsumoto K., McCulloch C.A., Yamada K.M., Larjava H. (2004). Glycogen Synthase Kinase-3 Regulates Cytoskeleton and Translocation of Rac1 in Long Cellular Extensions of Human Keratinocytes. Exp. Cell Res..

[77] Salinas P.C. (2005). Retrograde Signalling at the Synapse: A Role for Wnt Proteins. In Proceedings of the Biochemical Society Transactions. Biochem. Soc. Trans..

[78] Lucas F.R., Goold R.G., Gordon-Weeks P.R., Salinas P.C. (1998). Inhibition of GSK-3β Leading to the Loss of Phosphorylated MAP-1B Is an Early Event in Axonal Remodelling Induced by WNT-7a or Lithium. J. Cell Sci..

[79] Hall A.C., Lucas F.R., Salinas P.C. (2000). Axonal Remodeling and Synaptic Differentiation in the Cerebellum Is Regulated by WNT-7a Signaling. Cell.

[80] Chin L.S., Li L., Ferreira A., Kosik K.S., Greengard P. (1995). Impairment of Axonal Development and of Synaptogenesis in Hippocampal Neurons of Synapsin I-Deficient Mice. Proc. Natl. Acad. Sci. USA.

[81] Rosahl T.W., Spillane D., Missler M., Herz J., Selig D.K., Wolff J.R., Hammer R.E., Malenka R.C., Südhof T.C. (1995). Essential Functions of Synapsins I and II in Synaptic Vesicle Regulation. Nature.

[82] Lucas F.R., Salinas P.C. (1997). WNT-7a Induces Axonal Remodeling and Increases Synapsin I Levels in Cerebellar Neurons. Dev. Biol..

[83] Zhu L.Q., Wang S.H., Liu D., Yin Y.Y., Tian Q., Wang X.C., Wang Q., Chen J.G., Wang J.Z. (2007). Activation of Glycogen Synthase Kinase-3 Inhibits Long-Term Potentiation with Synapse-Associated Impairments. J. Neurosci..

[84] Hooper C., Markevich V., Plattner F., Killick R., Schofield E., Engel T., Hernandez F., Anderton B., Rosenblum K., Bliss T. (2007). Glycogen Synthase Kinase-3 Inhibition Is Integral to Long-Term Potentiation. Eur. J. Neurosci..

[85] Greengard P., Valtorta F., Czernik A.J., Benfenati F. (1993). Synaptic Vesicle Phosphoproteins and Regulation of Synaptic Function. Science.

[86] Chen J., Chang S.P., Tang S.J. (2006). Activity-Dependent Synaptic Wnt Release Regulates Hippocampal Long Term Potentiation. J. Biol. Chem..

[87] Opazo P., Watabe A.M., Grant S.G.N., O’Dell T.J. (2003). Phosphatidylinositol 3-Kinase Regulates the Induction of Long-Term Potentiation through Extracellular Signal-Related Kinase-Independent Mechanisms. J. Neurosci..

[88] Clayton E.L., Sue N., Smillie K.J., O’Leary T., Bache N., Cheung G., Cole A.R., Wyllie D.J., Sutherland C., Robinson P.J. (2010). Dynamin i Phosphorylation by GSK3 Controls Activity-Dependent Bulk Endocytosis of Synaptic Vesicles. Nat. Neurosci..

[89] La T.M., Tachibana H., Li S.A., Abe T., Seiriki S., Nagaoka H., Takashima E., Takeda T., Ogawa D., Makino S. (2020). Dynamin 1 Is Important for Microtubule Organization and Stabilization in Glomerular Podocytes. FASEB J..

[90] Šamaj J., Baluška F., Voigt B., Schlicht M., Volkmann D., Menzel D. (2004). Endocytosis, Actin Cytoskeleton, and Signaling. Plant. Physiol..

[91] Lossi L., Merighi A. (2003). In Vivo Cellular and Molecular Mechanisms of Neuronal Apoptosis in the Mammalian CNS. Prog. Neurobiol..

[92] Gibson R.M. (2001). Does Apoptosis Have a Role in Neurodegeneration?. Br. Med. J..

[93] D’Mello S.R., Anelli R., Calissano P. (1994). Lithium Induces Apoptosis in Immature Cerebellar Granule Cells but Promotes Survival of Mature Neurons. Exp. Cell Res..

[94] Hetman M., Cavanaugh J.E., Kimelman D., Zhengui X. (2000). Role of Glycogen Synthase Kinase-3β in Neuronal Apoptosis Induced by Trophic Withdrawal. J. Neurosci..

[95] Brewster J.L., Linseman D.A., Bouchard R.J., Loucks F.A., Precht T.A., Esch E.A., Heidenreich K.A. (2006). Endoplasmic Reticulum Stress and Trophic Factor Withdrawal Activate Distinct Signaling Cascades That Induce Glycogen Synthase Kinase-3β and a Caspase-9-Dependent Apoptosis in Cerebellar Granule Neurons. Mol. Cell. Neurosci..

[96] Lee K.Y., Koh S.H., Noh M.Y., Park K.W., Lee Y.J., Kim S.H. (2007). Glycogen Synthase Kinase-3β Activity Plays Very Important Roles in Determining the Fate of Oxidative Stress-Inflicted Neuronal Cells. Brain Res..

[97] Tsukane M., Yoshizaki C., Yamauchi T. (2007). Development and Specific Induction of Apoptosis of Cultured Cell Models Overexpressing Human Tau during Neural Differentiation: Implication in Alzheimer’s Disease. Anal. Biochem..

[98] Jope R.S., Yuskaitis C.J., Beurel E. (2007). Glycogen Synthase Kinase-3 (GSK3): Inflammation, Diseases, and Therapeutics. Neurochem. Res..

[99] Yuskaitis C.J., Jope R.S. (2009). Glycogen Synthase Kinase-3 Regulates Microglial Migration, Inflammation, and Inflammation-Induced Neurotoxicity. Cell. Signal..

[100] Nichols M.R., St-Pierre M.K., Wendeln A.C., Makoni N.J., Gouwens L.K., Garrad E.C., Sohrabi M., Neher J.J., Tremblay M.E., Combs C.K. (2019). Inflammatory Mechanisms in Neurodegeneration. J. Neurochem..

[101] Ko C.Y., Wang W.L., Wang S.M., Chu Y.Y., Chang W.C., Wang J.M. (2014). Glycogen Synthase Kinase-3β-Mediated CCAAT/Enhancer-Binding Protein Delta Phosphorylation in Astrocytes Promot Es Migration and Activation of Microglia/Macrophages. Neurobiol. Aging..

[102] Paul C.D., Mistriotis P., Konstantopoulos K. (2017). Cancer cell motility: Lessons from migration in confined spaces. Nat. Rev. Cancer..

[103] Farooqui R., Zhu S., Fenteany G. (2006). Glycogen synthase kinase-3 acts upstream of ADP-ribosylation factor 6 and Rac1 to regulate epithelial cell migration. Exp. Cell Res..

[104] Chikano Y., Domoto T., Furuta T., Sabit H., Kitano-Tamura A., Pyko I.V., Takino T., Sai Y., Hayashi Y., Sato H. (2015). Glycogen synthase kinase 3β sustains invasion of glioblastoma via the focal adhesion kinase, Rac1, and c-Jun N-terminal kinase-mediated pathway. Mol. Cancer Ther..

[105] Kitano A., Shimasaki S., Chikano Y., Nakada M., Hirose M., Higashi T., Ishigaki Y., Endo Y., Takino T., Sato H. (2013). Aberrant glycogen synthase kinase 3β is involved in pancreatic cancer cell invasion and resistance to therapy. PLoS ONE.

[106] Wang W.L., Hong T.M., Chang Y.L., Wu C.T., Pan S.H., Yang P.C. (2012). Phosphorylation of LCRMP-1 by GSK3β promotes filopoda formation, migration and invasion abilities in lung cancer cells. PLoS ONE.

[107] Le Grand M., Rovini A., Bourgarel-Rey V., Honore S., Bastonero S., Braguer D., Carre M. (2014). ROS-mediated EB1 phosphorylation through Akt/GSK3β pathway: Implication in cancer cell response to microtubule-targeting agents. Oncotarget.

[108] Kim J.G., Kim M.J., Choi W.J., Moon M.Y., Kim H.J., Lee J.Y., Kim J., Kim S.C., Kang S.G., Seo G.Y. (2017). Wnt3A Induces GSK-3β Phosphorylation and β-Catenin Accumulation Through RhoA/ROCK. J. Cell Physiol..

[109] Park S.Y., Lee Y.K., Lee W.S., Park O.J., Kim Y.M. (2014). The involvement of AMPK/GSK3-beta signals in the control of metastasis and proliferation in hepato-carcinoma cells treated with anthocyanins extracted from Korea wild berry Meoru. BMC Complement. Altern. Med..

[110] Parri M., Chiarugi P. (2010). Rac and Rho GTPases in cancer cell motility control. Cell Commun. Signal..

[111] Schaks M., Giannone G., Rottner K. (2019). Actin dynamics in cell migration. Essays Biochem..

[112] Yamazaki D., Kurisu S., Takenawa T. (2009). Involvement of Rac and Rho signaling in cancer cell motility in 3D substrates. Oncogene..

[113] Zhou T., Wang C.H., Yan H., Zhang R., Zhao J.B., Qian C.F., Xiao H., Liu H.Y. (2016). Inhibition of the Rac1-WAVE2-Arp2/3 signaling pathway promotes radiosensitivity via downregulation of cofilin-1 in U251 human glioma cells. Mol. Med. Rep..

[114] To C., Roy A., Chan E., Prado M.A.M., Di Guglielmo G.M. (2017). Synthetic triterpenoids inhibit GSK3β activity and localization and affect focal adhesions and cell migration. Biochim Biophys Acta Mol. Cell Res..

[115] To C., Shilton B.H., Di Guglielmo G.M. (2010). Synthetic triterpenoids target the Arp2/3 complex and inhibit branched actin polymerization. J. Biol Chem..

[116] Rom S., Fan S., Reichenbach N., Dykstra H., Ramirez S.H., Persidsky Y. (2012). Glycogen synthase kinase 3β inhibition prevents monocyte migration across brain endothelial cells via Rac1-GTPase suppression and down-regulation of active integrin conformation. Am. J. Pathol..

[117] Yoshino Y., Suzuki M., Takahashi H., Ishioka C. (2015). Inhibition of invasion by glycogen synthase kinase-3 beta inhibitors through dysregulation of actin re-organisation via down-regulation of WAVE2. Biochem Biophys Res. Commun..

[118] Seiz J.R., Klinke J., Scharlibbe L., Lohfink D., Heipel M., Ungefroren H., Giehl K., Menke A. (2020). Different signaling and functionality of Rac1 and Rac1b in the progression of lung adenocarcinoma. Biol Chem..

[119] Suraneni P., Fogelson B., Rubinstein B., Noguera P., Volkmann N., Hanein D., Mogilner A., Li R. (2015). A mechanism of leading-edge protrusion in the absence of Arp2/3 complex. Mol. Biol Cell..

[120] Jacquemet G., Hamidi H., Ivaska J. (2015). Filopodia in cell adhesion, 3D migration and cancer cell invasion. Curr Opin Cell Biol..

[121] Pan S.H., Chao Y.C., Chen H.Y., Hung P.F., Lin P.Y., Lin C.W., Chang Y.L., Wu C.T., Lee Y.C., Yang S.C. (2010). Long form collapsin response mediator protein-1 (LCRMP-1) expression is associated with clinical outcome and lymph node metastasis in non-small cell lung cancer patients. Lung Cancer..

[122] Krugmann S., Jordens I., Gevaert K., Driessens M., Vandekerckhove J., Hall A. (2001). Cdc42 induces filopodia by promoting the formation of an IRSp53:Mena complex. Curr. Biol..

[123] Schoumacher M., Goldman R.D., Louvard D., Vignjevic D.M. (2010). Actin, microtubules, and vimentin intermediate filaments cooperate for elongation of invadopodia. J. Cell Biol..

[124] Abe K., Yamamoto N., Domoto T., Bolidong D., Hayashi K., Takeuchi A., Miwa S., Igarashi K., Inatani H., Aoki Y. (2020). Glycogen synthase kinase 3β as a potential therapeutic target in synovial sarcoma and fibrosarcoma. Cancer Sci..

[125] Ying X., Jing L., Ma S., Li Q., Luo X., Pan Z., Feng Y., Feng P. (2015). GSK3β mediates pancreatic cancer cell invasion in vitro via the CXCR4/MMP-2 Pathway. Cancer Cell Int..

[126] Mai W., Kong L., Yu H., Bao J., Song C., Qu G. (2021). Glycogen synthase kinase 3β promotes osteosarcoma invasion and migration via regulating PTEN and phosphorylation of focal adhesion kinase. Biosci Rep..

[127] Eddy R.J., Weidmann M.D., Sharma V.P., Condeelis J.S. (2017). Tumor Cell Invadopodia: Invasive Protrusions that Orchestrate Metastasis. Trends Cell Biol..

[128] Paluch E.K., Raz E. (2013). The role and regulation of blebs in cell migration. Curr. Opin. Cell Biol..

[129] Liu J., Zhang Y., Xu R., Du J., Hu Z., Yang L., Chen Y., Zhu Y., Gu L. (2013). PI3K/Akt-dependent phosphorylation of GSK3β and activation of RhoA regulate Wnt5a-induced gastric cancer cell migration. Cell. Signal..

[130] Kremenevskaja N., von Wasielewski R., Rao A.S., Schöfl C., Andersson T., Brabant G. (2005). Wnt-5a has tumor suppressor activity in thyroid carcinoma. Oncogene.

[131] Islam R., Kim J.G., Park Y., Cho J.Y., Cap K.C., Kho A.R. (2019). Chung WS, Suh SW, Park JB. Insulin induces phosphorylation of pyruvate dehydrogenase through RhoA activation pathway in HepG2 cells. FASEB J..

[132] Jiang W., Betson M., Mulloy R., Foster R., Lévay M., Ligeti E., Settleman J. (2008). p190A RhoGAP is a glycogen synthase kinase-3-beta substrate required for polarized cell migration. J. Biol. Chem..

[133] Etienne-Manneville S. (2013). Microtubules in cell migration. Annu. Rev. Cell Dev. Biol..

[134] Etienne-Manneville S. (2009). APC in cell migration. Adv. Exp. Med. Biol..

[135] Zaoui K., Honoré S., Isnardon D., Braguer D., Badache A. (2008). Memo-RhoA-mDia1 signaling controls microtubules, the actin network, and adhesion site formation in migrating cells. J. Cell Biol..

[136] Wen Y., Eng C.H., Schmoranzer J., Cabrera-Poch N., Morris E.J., Chen M., Wallar B.J., Alberts A.S., Gundersen G.G. (2004). EB1 and APC bind to mDia to stabilize microtubules downstream of Rho and promote cell migration. Nat. Cell Biol..

[137] Zhang L., Shay J.W. (2017). Multiple Roles of APC and its Therapeutic Implications in Colorectal Cancer. J. Natl. Cancer Inst..

[138] Chiu C.T., Liao C.K., Shen C.C., Tang T.K., Jow G.M., Wang H.S., Wu J.C. (2015). HYS-32-Induced Microtubule Catastrophes in Rat Astrocytes Involves the PI3K-GSK3beta Signaling Pathway. PLoS ONE.

[139] Nehlig A., Molina A., Rodrigues-Ferreira S., Honoré S., Nahmias C. (2017). Regulation of end-binding protein EB1 in the control of microtubule dynamics. Cell Mol. Life Sci..

[140] Dong B., Zhang S.S., Gao W., Su H., Chen J., Jin F., Bhargava A., Chen X., Jorgensen L., Alberts A.S. (2013). Mammalian diaphanous-related formin 1 regulates GSK3β-dependent microtubule dynamics required for T cell migratory polarization. PLoS ONE.

[141] Wittmann T., Waterman-Storer C.M. (2005). Spatial regulation of CLASP affinity for microtubules by Rac1 and GSK3beta in migrating epithelial cells. J. Cell Biol..

[142] Zaoui K., Benseddik K., Daou P., Salaün D., Badache A. (2010). ErbB2 receptor controls microtubule capture by recruiting ACF7 to the plasma membrane of migrating cells. Proc. Natl. Acad. Sci. USA.

[143] Kumar P., Lyle K.S., Gierke S., Matov A., Danuser G., Wittmann T. (2009). GSK3beta phosphorylation modulates CLASP-microtubule association and lamella microtubule attachment. J. Cell Biol..

[144] Wu X., Shen Q.T., Oristian D.S., Lu C.P., Zheng Q., Wang H.W., Fuchs E. (2011). Skin stem cells orchestrate directional migration by regulating microtubule-ACF7 connections through GSK3β. Cell.

[145] Kanchanawong P., Shtengel G., Pasapera A.M., Ramko E.B., Davidson M.W., Hess H.F., Waterman C.M. (2010). Nanoscale architecture of integrin-based cell adhesions. Nature.

[146] Schlaepfer D.D., Mitra S.K. (2004). Multiple connections link FAK to cell motility and invasion. Curr. Opin. Genet. Dev..

[147] Hu Y.L., Lu S., Szeto K.W., Sun J., Wang Y., Lasheras J.C., Chien S. (2014). FAK and paxillin dynamics at focal adhesions in the protrusions of migrating cells. Sci. Rep..

[148] Kobayashi T., Hino S., Oue N., Asahara T., Zollo M., Yasui W., Kikuchi A. (2006). Glycogen synthase kinase 3 and h-prune regulate cell migration by modulating focal adhesions. Mol. Cell Biol..

[149] John J.K., Paraiso K.H., Rebecca V.W., Cantini L.P., Abel E.V., Pagano N., Meggers E., Mathew R., Krepler C., Izumi V. (2012). GSK3β inhibition blocks melanoma cell/host interactions by downregulating N-cadherin expression and decreasing FAK phosphorylation. J. Investig. Dermatol..

[150] Cai X., Li M., Vrana J., Schaller M.D. (2006). Glycogen synthase kinase 3- and extracellular signal-regulated kinase-dependent phosphorylation of paxillin regulates cytoskeletal rearrangement. Mol. Cell Biol..

[151] Xu W., Ge Y., Liu Z., Gong R. (2014). Glycogen synthase kinase 3β dictates podocyte motility and focal adhesion turnover by modulating paxillin activity: Implications for the protective effect of low-dose lithium in podocytopathy. Am. J. Pathol..

[152] Nayal A., Webb D.J., Brown C.M., Schaefer E.M., Vicente-Manzanares M., Horwitz A.R. (2006). Paxillin phosphorylation at Ser273 localizes a GIT1-PIX-PAK complex and regulates adhesion and protrusion dynamics. J. Cell Biol..

[153] Juanes M.A., Isnardon D., Badache A., Brasselet S., Mavrakis M., Goode B.L. (2019). The role of APC-mediated actin assembly in microtubule capture and focal adhesion turnover. J. Cell Biol..

[154] Wu X., Kodama A., Fuchs E. (2008). ACF7 regulates cytoskeletal-focal adhesion dynamics and migration and has ATPase activity. Cell.

[155] Yue J., Zhang Y., Liang W.G., Gou X., Lee P., Liu H., Lyu W., Tang W.J., Chen S.Y., Yang F. (2016). In vivo epidermal migration requires focal adhesion targeting of ACF7. Nat. Commun..

[156] Zhang H., Schaefer A., Wang Y., Hodge R.G., Blake D.R., Diehl J.N., Papageorge A.G., Stachler M.D., Liao J., Zhou J. (2020). Gain-of-Function RHOA Mutations Promote Focal Adhesion Kinase Activation and Dependency in Diffuse Gastric Cancer. Cancer Discov..

[157] Zhao J., Zhang J., Yu M., Xie Y., Huang Y., Wolff D.W., Abel P.W., Tu Y. (2013). Mitochondrial dynamics regulates migration and invasion of breast cancer cells. Oncogene.

[158] Tang J., Oliveros A., Jang M.H. (2019). Dysfunctional mitochondrial bioenergetics and synaptic degeneration in Alzheimer disease. Int. Neurourol. J..

[159] Chinnery P.F., Adam M.P., Ardinger H.H., Pagon R.A., Wallace S.E. (2014). Mitochondrial Disorders Overview. GeneReviews^®^.

[160] Tilokani L., Nagashima S., Paupe V., Prudent J. (2018). Mitochondrial dynamics: Overview of molecular mechanisms. Essays Biochem..

[161] Dixit R., Ross J.L., Goldman Y.E., Holzbaur E.L.F. (2008). Differential regulation of dynein and kinesin motor proteins by tau. Science.

[162] Lovas J.R., Wang X. (2013). The meaning of mitochondrial movement to a neuron’s life. Biochim. Biophys. Acta-Mol. Cell Res..

[163] Frederick R.L., Shaw J.M. (2007). Moving mitochondria: Establishing distribution of an essential organelle. Traffic.

[164] Deheshi S., Pasqualotto B.A., Rintoul G.L. (2013). Mitochondrial trafficking in neuropsychiatric diseases. Neurobiol. Dis..

[165] Altieri D.C. (2017). Mitochondria on the move: Emerging paradigms of organelle trafficking in tumour plasticity and metastasis. Br. J. Cancer.

[166] Shanmughapriya S., Langford D., Natarajaseenivasan K. (2020). Inter and Intracellular mitochondrial trafficking in health and disease. Ageing Res. Rev..

[167] Wang Q., Tian J., Chen H., Du H., Guo L. (2019). Amyloid beta-mediated KIF5A deficiency disrupts anterograde axonal mitochondrial movement. Neurobiol. Dis..

[168] Baas P.W., Qiang L. (2005). Neuronal microtubules: When the MAP is the roadblock. Trends Cell Biol..

[169] Kopeikina K.J., Carlson G.A., Pitstick R., Ludvigson A.E., Peters A., Luebke J.I., Koffie R.M., Frosch M.P., Hyman B.T., Spires-Jones T.L. (2011). Tau accumulation causes mitochondrial distribution deficits in neurons in a mouse model of tauopathy and in human Alzheimer’s disease brain. Am. J. Pathol..

[170] Morel M., Authelet M., Dedecker R., Brion J.P. (2010). Glycogen synthase kinase-3β and the p25 activator of cyclin dependent kinase 5 increase pausing of mitochondria in neurons. Neuroscience.

[171] Griebel G., Stemmelin J., Lopez-Grancha M., Boulay D., Boquet G., Slowinski F., Pichat P., Beeské S., Tanaka S., Mori A. (2019). The selective GSK3 inhibitor, SAR502250, displays neuroprotective activity and attenuates behavioral impairments in models of neuropsychiatric symptoms of Alzheimer’s disease in rodents. Sci. Rep..

[172] Cavendish J.Z., Sarkar S.N., Colantonio M.A., Quintana D.D., Ahmed N., White B.A., Engler-Chiurazzi E.B., Simpkins J.W. (2019). Mitochondrial Movement and Number Deficits in Embryonic Cortical Neurons from 3xTg-AD Mice. J. Alzheimer’s Dis..

[173] Llorens-Martín M., López-Doménech G., Soriano E., Avila J. (2011). GSK3β is involved in the relief of mitochondria pausing in a Tau-dependent manner. PLoS ONE.

[174] Emamian E.S., Hall D., Birnbaum M.J., Karayiorgou M., Gogos J.A. (2004). Convergent evidence for impaired AKT1-GSK3β signaling in schizophrenia. Nat. Genet..

[175] Chan M.H., Chiu P.H., Lin C.Y., Chen H.H. (2012). Inhibition of glycogen synthase kinase-3 attenuates psychotomimetic effects of ketamine. Schizophr. Res..

[176] Ogawa F., Murphy L.C., Malavasi E.L.V., O’Sullivan S.T., Torrance H.S., Porteous D.J., Millar J.K. (2016). NDE1 and GSK3β Associate with TRAK1 and Regulate Axonal Mitochondrial Motility: Identification of Cyclic AMP as a Novel Modulator of Axonal Mitochondrial Trafficking. ACS Chem. Neurosci..

[177] Emamian E.S. (2012). AKT/GSK3 signaling pathway and schizophrenia. Front. Mol. Neurosci..

[178] Kim J., Cheong J.H. (2020). Role of Mitochondria-Cytoskeleton Interactions in the Regulation of Mitochondrial Structure and Function in Cancer Stem Cells. Cells.

[179] Cunniff B., McKenzie A.J., Heintz N.H., Howe A.K. (2016). AMPK activity regulates trafficking of Mitochondria to the leading edge during cell migration and matrix invasion. Mol. Biol. Cell.

[180] Jiang N., Dai Q., Su X., Fu J., Feng X., Peng J. (2020). Role of PI3K/AKT pathway in cancer: The framework of malignant behavior. Mol. Biol. Rep..

[181] Caino M.C., Ghosh J.C., Chae Y.C., Vaira V., Rivadeneira D.B., Faversani A., Rampini P., Kossenkov A.V., Aird K.M., Zhang R. (2015). PI3K therapy reprograms mitochondrial trafficking to fuel tumor cell invasion. Proc. Natl. Acad. Sci. USA.

[182] Yi M., Weaver D., Hajnóczky G. (2004). Control of mitochondrial motility and distribution by the calcium signal: A homeostatic circuit. J. Cell Biol..

[183] Ramaccini D., Montoya-Uribe V., Aan F.J., Modesti L., Potes Y., Wieckowski M.R., Krga I., Glibetić M., Pinton P., Giorgi C. (2021). Mitochondrial Function and Dysfunction in Dilated Cardiomyopathy. Front. Cell. Dev. Biol..

[184] Viola H.M., Davies S.M., Filipovska A., Hool L.C. (2013). L-type Ca(2+) channel contributes to alterations in mitochondrial calcium handling in the mdx ventricular myocyte. Am. J. Physiol. Heart Circ. Physiol..

[185] Fernandez-Sanz C., Ruiz-Meana M., Miro-Casas E., Nuñez E., Castellano J., Loureiro M., Barba I., Poncelas M., Rodriguez-Sinovas A., Vázquez J. (2014). Defective sarcoplasmic reticulum-mitochondria calcium exchange in aged mouse myocardium. Cell Death Dis..

[186] Saotome M., Safiulina D., Szabadkai G., Das S., Fransson A., Aspenstrom P., Rizzuto R., Hajnóczky G. (2008). Bidirectional Ca2+-dependent control of mitochondrial dynamics by the Miro GTPase. Proc. Natl. Acad. Sci. USA.

[187] Mills K.M., Brocardo M.G., Henderson B.R. (2016). APC binds the Miro/Milton motor complex to stimulate transport of mitochondria to the plasma membrane. Mol. Biol. Cell.

