# Supplementary material for: GSK3 as a Regulator of Cytoskeleton Architecture: Consequences for Health and Disease
Source: Cells. 2021 Aug 14;10(8):2092. doi: 10.3390/cells10082092 (PMC8393567; doi:10.3390/cells10082092)
Supplement: Supplementary file 1 [file cells-10-02092-s001.zip › cells-1321287-supplementary/cells-1321287-SM/Supplementary tables/Supplementary Table S1.pdf]

Supplementary Table S1. Main (direct and indirect) GSK3 targets mentioned in the present paper, their cellular roles, major effects of the GSK3-target protein interactions, together with the experimental model and methods of detection used in the reviewed papers.

GSK3β target	Function of target protein	Effects of active GSK3β on target activity/expression	Experimental model	Detection method	Source
**Metabolic/signaling proteins**					
cyclin D1	cell cycle progression	expression promotion	rat primary cultures of cerebellar granule neurons	Western blot	[21]
cyclin E	cell cycle progression	expression promotion	rat primary cultures of cerebellar granule neurons	Western blot, immunocytochemistry	[21]
retinoblastoma protein	cell cycle progression, uncontrolled cell proliferation	positive regulation (phosphorylation, which occurs during PD)	rat primary cultures of cerebellar granule neurons	Western blot, immunocytochemistry	[21]
APC	microtubules assembly, cell migration, axon elongation	negative regulation	rat primary astrocyte cell culture	cell migration assay	[51]
			mouse embryonic dorsal root ganglia cell culture	immunocytochemistry	[61]
			mouse embryonic fibroblasts	immunocytochemistry	[140]
PTEN	stops axon branching	positive regulation	mouse dentate gyri	Western blot, immunohistochemistry	[68]
Rac1	cell migration	positive regulation	Madin-Darby Canine Kidney cells	Western blot	[103]
FAK	cell adhesion and migration	positive regulation	human glioblastoma cell lines (T98G, U87)	Western blot	[104]
			human pancreatic cell lines (MIA PaCa-2, PANC-1, BxPC-3)	Western blot, immunohistochemistry	[105]
			Rat2 fibroblasts	Western blot, immunoprecipation	[114]
			human osteosarcoma cell line (MG-63)	immunocytochemistry	[126]
			human melanoma cell lines (WM793, 1205Lu, WM9)	immunocytochemistry, mass spectrometry	[149]
WASF2	cell migration	positive regulation	human breast adenocarcinoma cell line (MDA-MB_231)	Western blot	[117]
TRAK1	mitochondrial anterograde movement	positive regulation (Gsk3β binds to TARK1)	embryonic human kidney cell line HEK 293, human neuroblast form neural tissue cell line SH-SY5Y	immunoprecipitation	[176]
			mouse primary cultures of hippocampal neurons	mitochondrial movement measurement	
**Structural proteins**					
Astrin	spindle organisation	allows Astrin to interact with microtubules and kinetochore	in vitro (yeast two-hybrid assay)	Western blot, immunocytochemistry	[22]
			human HeLa cell line	Western blot, immunocytochemistry	
Ninein	microtubules	inhibits Ninein	human tissues (heart,	PCR, Northern blot	[25]

	organisation during cell division	accumulation in centrosomes and promotes proliferation	brain, placenta, lung, liver, skeletal muscle, kidney, pancreas		
			in vitro (yeast two-hybrid assay)	Western blot	[26]
CRMP2	microtubules assembly, axon branching	negative regulation	human embryonic kidney cell line HEK293, human neuroblast form neural tissue cell line SH-SY5Y	SDS-PAGE, autoradiography, mass spectrometry	[60]
Arp2/3	dendritic spines development, actin cytoskeleton branching in lamellipodia	positive regulation	aneuploid immortal keratinocyte cell line from adult human skin HaCaT	immunocytochemistry	[76]
Dynamin I	synaptic vesicles endocytosis	positive regulation	rat primary neuronal cultures	Western blot, autoradiography, SDS-PAGE	[88]
MT1-MMP	tumor invasion	expression promotion	human glioblastoma cell lines (U87, U25, T98G)	Western blot, PCR	[104]
LCRMP-1	filopodia formation, cell migration, cancer invasion enhancer	positive regulation	primary human lung cancer cells	Western blot, protein sequences alignment	[106]
EB1	cell migration	negative regulation	human non-small lung carcinoma A549 cell line	Western blot, indirect immunofluorescence analysis	[107]
CLASP2	cell migration	negative regulation	human breast SkBr3 carcinoma cell line	Western blot, time-lapse fluorescence microscopy	[142]
			human keratinocyte HaCaT call line, human cervix epithelial HeLa cell line	Western blot, immunoprecipitation, SDS-PAGE	[143]
ACF7	actin binging protein, cell migration	negative regulation	hair follicle stem cells (HF-SC)	Western blot, SDS-PAGE, autoradiography	[144]
Paxillin	cell migration	positive regulation	RAW 264.7 mouse macrophages	Western blot	[150]
			embryonic human kidney cell line HEK 293, human neuroblast form neural tissue cell line SH-SY5Y	immunoprecipitation	[176]
Tau	microtubule assembly	negative regulation	mouse primary cultures of hippocampal neurons	immunocytochemistry, live-imaging and quantification of axonal transport of mitochondria	[173]
		**Transcription factors**			
E2F-1	cell cycle progression	expression promotion	rat primary cultures of cerebellar granule neurons	Western blot, PCR, immunocytochemistry	[21]
AP-1	differentiation, proliferation and apoptosis	negative regulation	JB6 P+ mouse epidermal cell line (Cl 41)	activity measurement	[27]
NF-κB	inflammatory processes, cell proliferation, differentiation and survival	negative regulation	rat primary cultures of cerebellar granule neurons	ELISA	[33]
			rat primary cultures of astrocytes	Western blot	[34]

β-catenin	cell proliferation and differentiation	negative regulation	human embryonic kidney cell line HEK293	Western blot	[35]
			cortical neurons	Western blot	[46]
MAP1B	axonal growth and regeneration	positive regulation	dorsal root ganglia cell culture	Western blot	[61]
CEBPD	microglia activation	positive regulation	human glioblastoma-astrocytoma cell line U373MG	Western blot, wound-healing migration assays, chemotaxis assay	[101]
