# Supplementary material for: GSK3 as a Regulator of Cytoskeleton Architecture: Consequences for Health and Disease
Source: Cells. 2021 Aug 14;10(8):2092. doi: 10.3390/cells10082092 (PMC8393567; doi:10.3390/cells10082092)
Supplement: Supplementary file 1 [file cells-10-02092-s001.zip › cells-1321287-supplementary/cells-1321287-SM/Supplementary tables/Supplementary Table S2.pdf]

**Supplementary Table S2.** Main upstream proteins/pathways regulating GSK3β activity mentioned in the present paper, cellular processes they stimulate, and their influence on the activity of GSK3β, together with the experimental model and methods of detection used in the reviewed papers.

| GSK3β regulators | Function of active protein/pathway | Effects of active protein/pathway on GSK3β activity | Experimental model | Detection method | Source |
|---|---|---|---|---|---|
| **PI3K/Akt** | cell differentiation | negative regulation | rat primary cultures of hippocampal neurons | Western blot, immunocytochemistry | [58] |
| | | | | Western blot | [59] |
| **RhoA** | cell migration | negative regulation | human HEPG2 cancer cell line | Western blot | [131] |
| **Cdc42/Par6/ PKCζ** | asymmetric cell division promoted by interactions of APC with the plus ends of microtubules | negative regulation | rat primary cultures of astrocytes | Western blot, immunocytochemistry, cell migration assay | [51] |
| **RhoGTPase/Cdc42/PKC** | prevention of actin branching and lamellipodia formation | negative regulation | rat primary cultures of astrocytes | Western blot, immunocytochemistry, cell migration assay | [51] |
| **ROCK1** | amoeboid movement stimulation | negative regulation | RAW 264.7 mouse macrophages, human embryonic kidney cell line HEK293 | Western blot | [108] |
| **ErbB2** | microtubules assembly, cell motility | negative regulation | Human Breast Cancer Cell Line SK-BR-3 | Western blot, immunocytochemistry, time-lapse fluorescence microscopy | [142] |
