# Supplementary material for: GSK3 as a Regulator of Cytoskeleton Architecture: Consequences for Health and Disease
Source: Cells. 2021 Aug 14;10(8):2092. doi: 10.3390/cells10082092 (PMC8393567; doi:10.3390/cells10082092)
Supplement: Supplementary file 1 [file cells-10-02092-s001.zip › cells-1321287-supplementary/Supplementary tables/Supplementary Table S1.pdf]

Supplementary Table S1. Main (direct and indirect) GSK3 targets mentioned in the present paper, their cellular roles, major effects of the GSK3-target protein interactions, together with the experimental model and methods of detection used in the reviewed papers.

GSK3β target	Function of target protein	Effects of active GSK3β on target activity/expression	Experimental model	Detection method	Source
		**Metabolic/signaling proteins**			
cyclin D1	cell cycle progression	expression promotion	rat primary cultures of cerebellar granule neurons	Western blot	[21]
cyclin E	cell cycle progression	expression promotion	rat primary cultures of cerebellar granule neurons	Western blot, immunocytochemistry	[21]
retinoblastoma protein	cell cycle progression, uncontrolled cell proliferation	positive regulation (phosphorylation, which occurs during PD)	rat primary cultures of cerebellar granule neurons	Western blot, immunocytochemistry	[21]
APC	microtubules assembly, cell migration, axon elongation	negative regulation	rat primary astrocyte cell culture	cell migration assay	[51]
			mouse embryonic dorsal root ganglia cell culture	immunocytochemistry	[61]
			mouse embryonic fibroblasts	immunocytochemistry	[140]
PTEN	stops axon branching	positive regulation	mouse dentate gyri	Western blot, immunohistochemistry	[68]
Rac1	cell migration	positive regulation	Madin-Darby Canine Kidney cells	Western blot	[103]
FAK	cell adhesion and migration	positive regulation	human glioblastoma cell lines (T98G, U87)	Western blot	[104]
			human pancreatic cell lines (MIA PaCa-2, PANC-1, BxPC-3)	Western blot, immunohistochemistry	[105]
			Rat2 fibroblasts	Western blot, immunoprecipation	[114]
			human osteosarcoma cell line (MG-63)	immunocytochemistry	[126]
			human melanoma cell lines (WM793, 1205Lu, WM9)	immunocytochemistry, mass spectrometry	[149]
WASF2	cell migration	positive regulation	human breast adenocarcinoma cell line (MDA-MB_231)	Western blot	[117]
TRAK1	mitochondrial anterograde movement	positive regulation (Gsk3β binds to TARK1)	embryonic human kidney cell line HEK 293, human neuroblast form neural tissue cell line SH-SY5Y	immunoprecipitation	[176]
			mouse primary cultures of hippocampal neurons	mitochondrial movement measurement	
		**Structural proteins**			
Astrin	spindle organisation	allows Astrin to interact with microtubules and kinetochore	in vitro (yeast two-hybrid assay)	Western blot, immunocytochemistry	[22]
			human HeLa cell line	Western blot, immunocytochemistry	
Ninein	microtubules organisation during cell division	inhibits Ninein accumulation in centrosomes and promotes proliferation	human tissues (heart, brain, placenta, lung, liver, skeletal muscle, kidney, pancreas	PCR, Northern blot	[25]
			in vitro (yeast two-hybrid assay)	Western blot	[26]

CRMP2	microtubules assembly, axon branching	negative regulation	human embryonic kidney cell line HEK293, human neuroblast form neural tissue cell line SH-SY5Y	SDS-PAGE, autoradiography, mass spectrometry	[60]
Arp2/3	dendritic spines development, actin cytoskeleton branching in lamellipodia	positive regulation	aneuploid immortal keratinocyte cell line from adult human skin HaCaT	immunocytochemistry	[76]
Dynamin I	synaptic vesicles endocytosis	positive regulation	rat primary neuronal cultures	Western blot, autoradiography, SDS-PAGE	[88]
MT1-MMP	tumor invasion	expression promotion	human glioblastoma cell lines (U87, U25, T98G)	Western blot, PCR	[104]
LCRMP-1	filopodia formation, cell migration, cancer invasion enhancer	positive regulation	primary human lung cancer cells	Western blot, protein sequences alignment	[106]
EB1	cell migration	negative regulation	human non-small lung carcinoma A549 cell line	Western blot, indirect immunofluorescence analysis	[107]
CLASP2	cell migration	negative regulation	human breast SkBr3 carcinoma cell line	Western blot, time-lapse fluorescence microscopy	[142]
			human keratinocyte HaCaT call line, human cervix epithelial HeLa cell line	Western blot, immunoprecipitation, SDS-PAGE	[143]
ACF7	actin binging protein, cell migration	negative regulation	hair follicle stem cells (HF-SC)	Western blot, SDS-PAGE, autoradiography	[144]
Paxillin	cell migration	positive regulation	RAW 264.7 mouse macrophages	Western blot	[150]
			embryonic human kidney cell line HEK 293, human neuroblast form neural tissue cell line SH-SY5Y	immunoprecipitation	[176]
Tau	microtubule assembly	negative regulation	mouse primary cultures of hippocampal neurons	immunocytochemistry, live-imaging and quantification of axonal transport of mitochondria	[173]
			**Transcription factors**		
E2F-1	cell cycle progression	expression promotion	rat primary cultures of cerebellar granule neurons	Western blot, PCR, immunocytochemistry	[21]
AP-1	differentiation, proliferation and apoptosis	negative regulation	JB6 P+ mouse epidermal cell line (Cl 41)	activity measurement	[27]
NF-κB	inflammatory processes, cell proliferation, differentiation and survival	negative regulation	rat primary cultures of cerebellar granule neurons	ELISA	[33]
			rat primary cultures of astrocytes	Western blot	[34]
β-catenin	cell proliferation and differentiation	negative regulation	human embryonic kidney cell line HEK293	Western blot	[35]

MAP1B	cell migration, axonal growth and regeneration	positive regulation	cortical neurons	Western blot	[46]
			mouse embryonic dorsal root ganglia cell culture	Western blot	[61]
CEBPD	microglia activation	positive regulation	human glioblastoma-astrocytoma cell line U373MG	Western blot, wound-healing migration assays, chemotaxis assay	[101]
